# Visualizing Cytoskeletal Protein Reconstruction of Vulvar Cancer with Surface-Enhanced Raman Spectroscopy and Gold Nanoparticles

**DOI:** 10.1021/acsomega.5c07659

**Published:** 2026-01-16

**Authors:** Kazushige Yokoyama, Kia Haering, Nicole Mathewson, Patrick Loss, Jani E. Lewis

**Affiliations:** † The State University of New York Geneseo College, Department of Chemistry and Biochemistry, Geneseo, New York 14454, United States; ‡ The State University of New York Geneseo College, Department of Biology, Geneseo, New York 14454, United States

## Abstract

Metastasis of epithelial cancers often involves epithelial–mesenchymal transition (EMT), characterized by changes in cytoskeletal and adhesion protein expression. This includes the loss of the cell adhesion protein E-cadherin and the gain of the cytoskeletal protein vimentin. Our laboratory found that the epithelial vulvar cancer cell line A431 undergoes permanent loss of E-cadherin and gains vimentin expression when treated with a corticosteroid known as clobetasol (referred to as A431D cells). Clobetasol is commonly used to treat chronic vulvar rashes, making our findings significant when considering the repercussions of this treatment. Interestingly, the cells continued to express cytokeratin 8/18 in addition to vimentin. Raman spectroscopy has been used to monitor EMT in breast and oral cancer. We used 3-dimensional Surface-Enhanced Raman Scattering (SERS) imaging by utilizing colloidal gold nanoparticles. By tracking protein interactions and surface composition in cells before and after treatment with clobetasol, the distribution of the transition can be reasoned and visualized. Spectral assignments revealed the order of protein gains and losses, while the distinctness of the collected signals from the literature signals provided information about the folding, binding, and repelling forces between these cytoskeletal proteins. The three-dimensional Raman imaging of the cytoplasmic region of A431D cells exhibited unique features that combined the cytoarchitecture of A431 cells (parent cells expressing cytokeratins 8 and 18) and NIH 3T3 cells (used because they only express vimentin). This suggests that Raman imaging can be used to delineate cells that contain multiple types of intermediate filaments and serve as another method to identify cells undergoing EMT.

## Graphical abstract

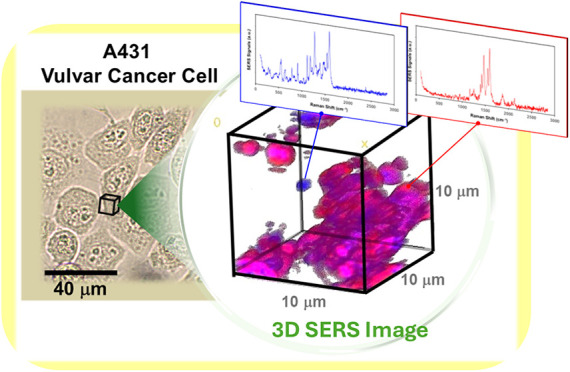

## Introduction

Intermediate filaments (IF) are a superfamily of 10 nm fibers present in most multicellular eukaryotes. Squamous epithelial cells are typically characterized by the expression of a subset of IF known as cytokeratins, composed of heteropolymers of type I and type II proteins.^1,2^ They also commonly express the transmembrane cell adhesion molecule epithelial (E) cadherin. Vimentin, a member of the type III IF family of proteins, is the major IF of mesenchymal cells and is involved in the migratory behavior of fibroblast cells during wound healing and repair. Cytokeratins and vimentin play key roles in cell adhesion, mechanical stability,^3–5^ cell migration, and intracellular signaling. Moreover, vimentin has been established as a marker of the epithelial to mesenchymal transition (EMT) in embryogenesis and in tumor metastasis.^6–9^

In addition to the gain of vimentin expression, the loss of E-cadherin expression is also a characteristic of EMT during cancer progression in epithelial tissues.^10,11^ These alterations are characteristic of the epithelial-to-mesenchymal transition (EMT), a process that occurs during both organogenesis and carcinogenesis.^12^ Several studies have shown that some cells undergoing EMT coexpress epithelial and mesenchymal markers, including dual expression of cytokeratins and vimentin.^13,14^ Furthermore, coexpression of cytokeratin and vimentin in some tumor types correlates with a worse prognosis than that seen for tumors expressing only cytokeratins.^15–19^

Vulvar squamous cell carcinoma (VSCC) is a rare malignancy, with an incidence of 2.6 per 100,000 women in the U.S.^20^ Patients with metastatic VSCC often face poor outcomes, particularly older women.^21^ Approximately 75% of VSCC cases originate from differentiated vulvar intraepithelial neoplasia (dVIN), commonly associated with vulvar lichen sclerosus (VLS), while the remaining 25% are linked to HPV-related high-grade squamous intraepithelial lesions (HSIL).^22^ Ultrapotent topical glucocorticoids, such as clobetasol, are the first-line treatment for VLS and are considered safe with long-term use.^23^ However, emerging evidence suggests that ultrapotent glucocorticoids (such as clobetasol) may induce cancer cell dormancy in other malignancies, potentially contributing to treatment resistance and recurrence.^24,25^ We have found that clobetasol treatment of the vulvar cancer cell line, A431, resulted in the initial loss of E-cadherin, followed by the gain of vimentin expression. Our system provides an inducible model to study this process. During clobetasol-induced EMT in the A431 cells, the expression of vimentin increases, but the cells do not lose the expression of cytokeratin molecules (cytokeratins 8 and 18). The transition of A431 cells occurs heterogeneously, with small patches of E-cadherin-negative cells arising 3–4 days after the addition of clobetasol, followed by an increase in vimentin expression after an additional 2–4 weeks. Some of the clobetasol-treated A431 populations never showed downregulation of E-cadherin and gain of vimentin expression. For these studies, we used a subcloned population of A431 cells that no longer expressed E-cadherin but showed dual expression of cytokeratin 8/18 and vimentin (referred to as A431D cells). Raman spectroscopy was used to identify cell state transitions, including EMT in cancer cells.^16,17^ We hypothesized that Raman spectroscopy could be used to distinguish between cells expressing cytokeratin 8/18 (A431) versus those coexpressing cytokeratin 8/18 and vimentin (A431D). We used a three-dimensional surface-enhanced Raman scattering (SERS) spectrum and Raman imaging paired with colloidal gold nanoparticles. This initial study identified distinct 3D morphological features of the pretreated (parent) cells, A431, compared to the postclobetasol-treated A431D cells. Raman imaging can be used to identify cytokeratins 8 and 18 and vimentin in live cells. This can then be used to “dissect out” the localization of these molecules in real time. Future studies will determine if Raman imaging can detect the earliest time points when vimentin expression increases and thus serve as an early detection method for the EMT, which is indicative of cancer progression. This can then be correlated by other means with molecular changes occurring with clobetasol treatment.

Few experimental methods allow the analysis of protein structure within cells.^26,27^ Förster resonance energy transfer (FRET) sensors can be used to selectively measure intramolecular distances that can be correlated to the tension acting on a target protein.^28,29^ However, this approach is challenging to realize in bundle-forming filaments, where cross-talk between neighboring dye molecules complicates the interpretation of the FRET signal.^30,31^ Cys-shotgun labeling of exposed cysteines is another method, introduced by Discher and colleagues, to show that IF proteins undergo conformational changes in response to cell tension by the exposure of otherwise buried cysteines.^32,33^ However, both FRET measurements and Cys-shotgun labeling are unable to probe a protein’s secondary structure, which is vital to rationalizing whether the *in vitro* load-bearing mechanism involving structural transitions is relevant for intracellular IF networks. The secondary structure of proteins can be probed in a noninvasive way using vibrational spectroscopies, such as infrared or Raman spectroscopy.^34^ Depending on the local hydrogen-bonding pattern (*i.e*., secondary structure), the atoms in the protein backbone (C=O vibrations in peptide bonds) exhibit molecular vibrations at different vibrational frequencies, resulting in shifted Raman peaks in the Amide I region of the vibrational spectrum.^35–37^ This link between the protein structure and spectral response can be exploited to determine the average secondary structural composition within the probed volume with high accuracy using quantitative spectral decomposition. In this study, we investigated if tension-induced secondary structural transitions of vimentin IF can be observed in adherent cells using SERS spectroscopy and Raman imaging with submicrometer spatial resolution to resolve the minimum cell structure unit in different tensile states.

## Results and Discussion

A431D cells were generated by treating A431 cells with dexamethasone (dex), as previously described.^7^ Dex-treated A431 cells caused some cells to lose the expression of E- and P-cadherin. These cells were subcloned, and it was found that they maintained the loss of E- and P-cadherin even in the absence of dex treatment. They were named A431D cells to distinguish them from the parental A431 cells. Subsequent studies found that treatment of A431 cells with the dex analog, clobetasol (clob), also resulted in A431D cells (Figure 1a). A431D cells display a loss of E-cadherin expression and a gain of vimentin expression (compare A431 to A431D, Figure 1b­(D,E)). The A431D cells, however, did not lose the expression of cytokeratin 8 (compare A431 to A431D, Figure 1b­(A,B), respectively) and cytokeratin 18 (Figure 1a). The distribution of vimentin was also visualized in the mouse fibroblast cell line, NIH-3T3, for comparison, since this cell line only expresses vimentin and not cytokeratin 8 (Figure 1b­(C,F), respectively). The cytoarchitecture of A431D cells changed with expression of vimentin, becoming more fibroblast-like in morphology (Figure 1b­(B,E)). Previous work showed that exogenous expression of E-cadherin in A431D cells did not alter the fibroblastic appearance of these cells^7^; however, these experiments did not measure the levels of vimentin expression. Future experiments will establish whether exogenous expression of E-cadherin in A431D cells expressing vimentin does not alter the fibroblastic appearance of these cells compared to that of the parent A431 cells.

**Figure 1:**
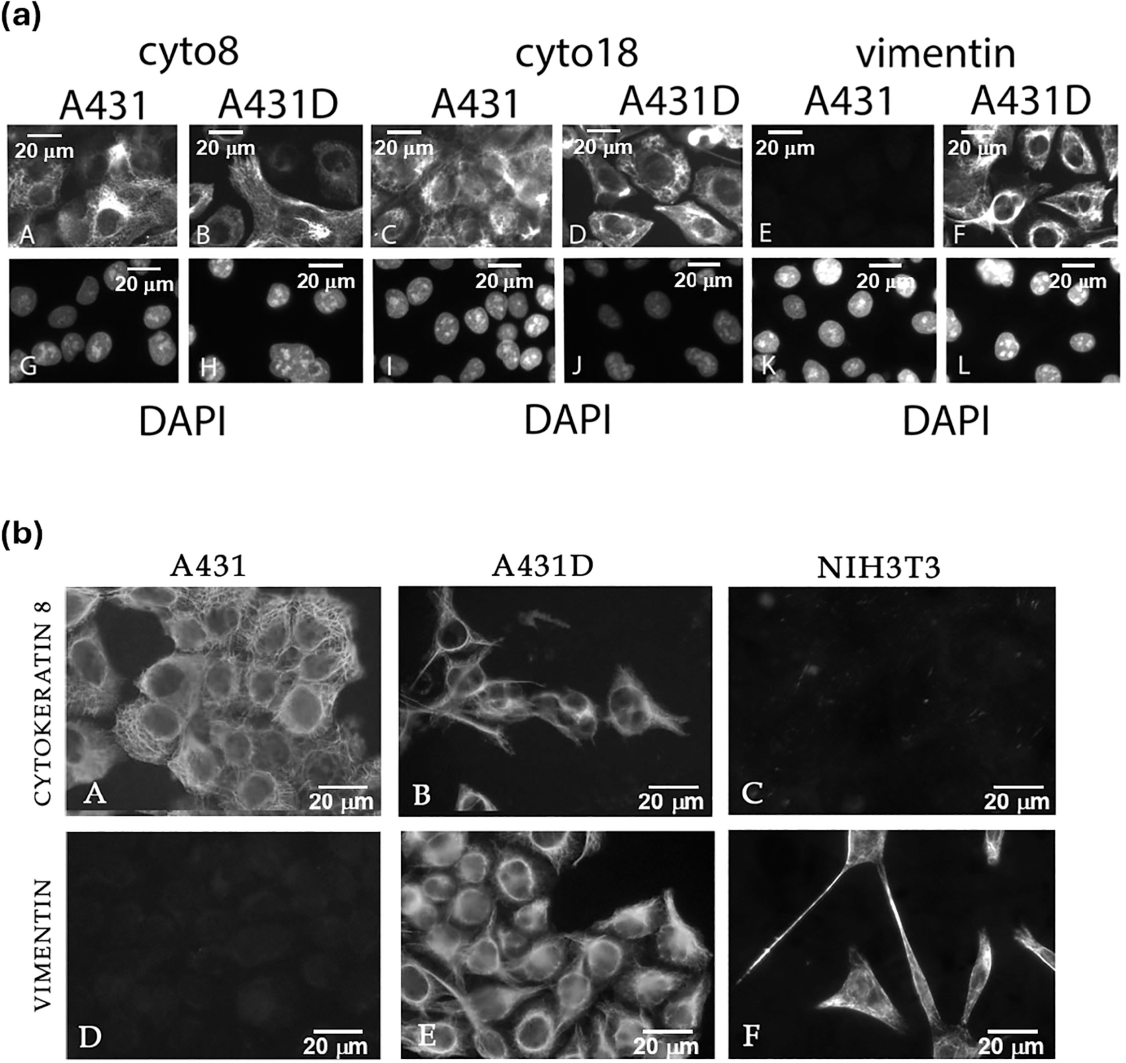
(a) A431 and A431D cells express cytokeratin 8 and 18 (panels A–D), respectively, whereas only A431D cells express vimentin (compare panels E and F). Panels G–L show the same cells as in panels A–F, but stained with DAPI to indicate the presence of nuclei. DAPI stained the nuclei of all cells. The exposure times for panels A–F were constant. (b) Immunofluorescence imaging of cytokeratin 8 expression shows that A431D cells did not lose cytokeratin 8 expression despite the upregulation of vimentin. Cytokeratin 8 localization in A431 (A), A431D (B), and NIH3T3 cells (C). Expression of vimentin in A431 (D), A431D (E), and NIH3T3 cells (F). NIH 3T3 cells expressed only vimentin and were stained with cytokeratin 8 and vimentin for comparison.

Following immunofluorescence analysis, we applied Raman imaging to obtain label-free, molecular-level structural information from the cells, providing complementary insights into their biochemical compositions. As a preparation, white-light images of A431, A431D, and NIH cells mixed with gold nanoparticles were observed at ×10, ×50, and ×100 magnifications (Figure 2). The bottom images correspond to the red-squared region of the ×100 magnification image. The regions marked by red-square boxes in the middle row sections in Figure 2 focus on the cytoplasm regions and were targeted in this work in the Raman imaging study.

**Figure 2:**
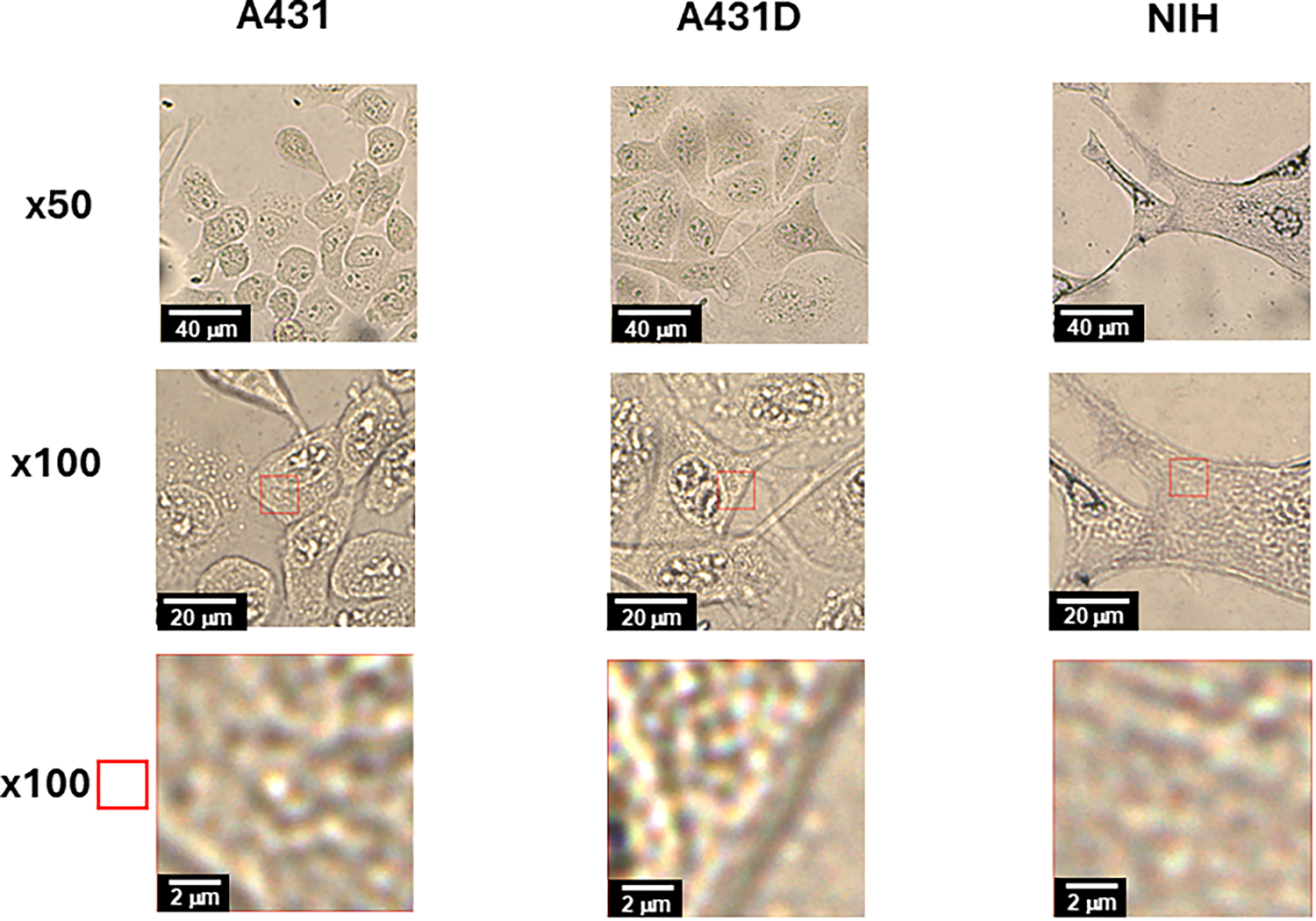
White-light images of A431, A431D, and NIH cells mixed with gold nanoparticles, observed at ×50 and ×100 magnifications. The bottom image corresponds to an enlargement of the red box in the ×100 magnification image above.

Centering the 10 μm × 10 μm red-square area shown in Figure 2, three-dimensional Raman images were collected for a cubical volume defined by 10 μm × 10 μm × 10 μm (Figure 3). For each Raman imaging analysis, most of the two spectral components were limited in order to avoid complications in the analysis. The distinction between the two spectral components was made using the following strategy. Component 1 mainly includes the fingerprint spectral region (Amide I, II, III bands, 1250–1750 cm^–1^), while Component 2 includes the fingerprint region as well as the lower-wavenumber side (200–1100 cm^–1^). A representative frame shot of the three-dimensional images based on these components was constructed, as shown on the left side of Figure 3. These three-dimensional images clearly show the unique and different morphologies of the cytoplasmic region. The representative SERS spectra of each cell are shown on the right side of Figure 3. The color of the 3D image in Figure 3 corresponds to the spectral component (*i.e*., Component 1 in red, Component 2 in blue, and a combination of the two components in purple). The spectral assignments of all the spectral lines observed for each component are shown in Table S1. The A431 cells exhibited a clump-like morphology, A431D occupied most of the space region, combined with clump and fiber-like forms, and the NIH cells showed almost fiber-like forms. Here, the combination of two spectral components, component 1 (red) and component 2 (blue), appears as purple.

**Figure 3:**
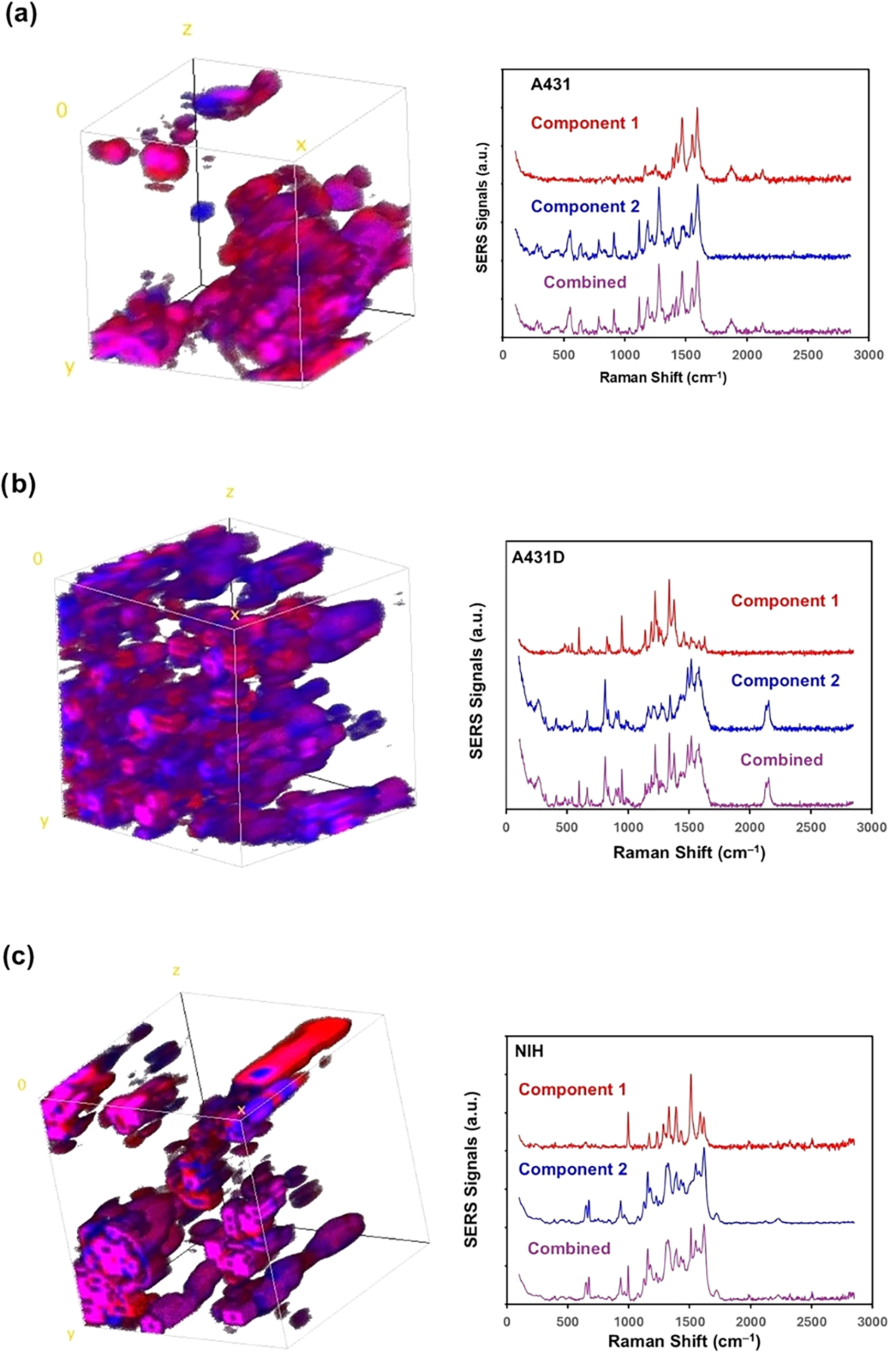
Representative frame view of three-dimensional Raman images (10 μm × 10 μm × 10 μm) of (a) A431, (b) A431D, and (c) NIH cells with the SERS spectrum (on the right) of the corresponding component shown in red and blue.

The combined SERS spectra of the two components are displayed with representative assignments in Figure 4 for (a) A431, (b) A431D, and (c) NIH cells, and the assignments of the spectral lines are listed in Table 1. The major Raman spectral lines (*ν̃*_obs_) in cm^–1^ are selected for (i) A431 Component 1, (ii) A431 Component 2, (iii) A431D Component 1, (iv) A431D Component 2, (v) NIH Component 1, and (vi) NIH Component 2. The detailed assignments are summarized in Table S1. A major spectral component in the fingerprint region was attributed to RNA (G, C, A, T) and proteins (Phe, Tyr, and Trp).

**Figure 4:**
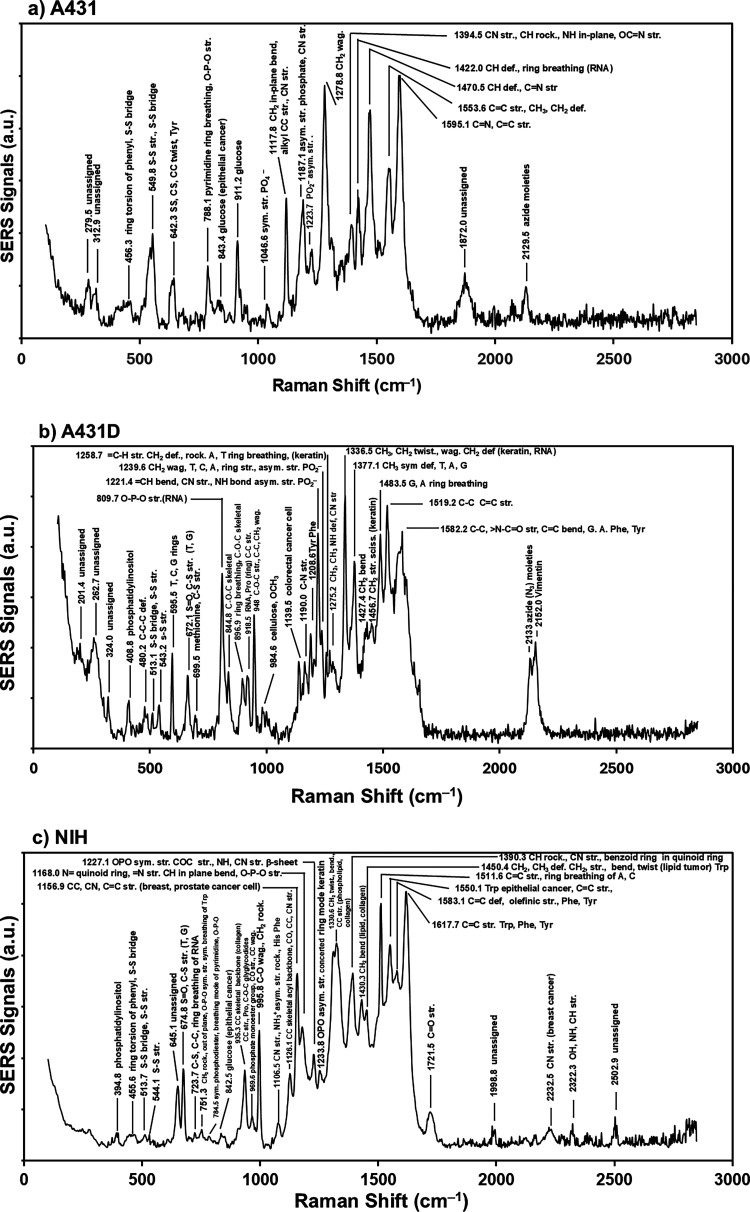
Two components combined SERS spectra of the (a) A431, (b) A431D, (c) NIH cells with spectral assignments.

**Table 1:** Major Raman Spectral Lines (*ν̃*_obs_) in cm^–1^ Selected for Combined Spectra of the (i) A431, (ii) A431D, and (iii) NIH Cells^a^

(i)A431		
(*ν̃*_obs_)		Assignment (reported *ν̃*_obs_)
**279.5**		unassigned
**312.9**	**⊕**	unassigned
**456.3**	**⊕**	ring torsion of phenyl-2,^44,53^ S–S bridge^54^
**549.8**	**⊕**	S–S str,^55^ S–S bridge^54^
**642.3**	**⊕**	C–S str., C–C twist.-Tyr^53,38,53,40–42^
**788.1**	**⊕**	pyrimidine ring breathing mode^53,56^ O–P–O str.^43,45^
**802.8**		backbone geometry and phosphate ion interactions^53,38^ C–C str. (lipids/fatty acids)^44^ C–H wag.^44^ Uracil-based ring breathing mode^53,56^
**843.4**		glucose (epithelial cancer)^40,53^
**911.2**	**⊕**	glucose^53,57^
**1046.6**	**⊕**	sym. str. of PO_4_®,^53,41^ glycogen^58,53,48,59^
**1117.8**	**⊕**	CH_2,6_ in-plane bend,^44,53^ alkyl C–C str. (Lipid),^60^ glucose,^53,57^ C–C str. (breast lipid) (epithelial cancer),^40,53^ CN str.,^51^ Pro (human breast tissue)^61^
**1167.2**		C–C str.,^44^ Pro (human breast tissue),^61^ *N* = str. quinoid ring, = =N str. and C–H in-plane bend,^53,62^ lipids,^53,63^ C=C str. def. COH (lipid),^53,64^ C–C str.,^53,65^ Tyr (collagen)^53,41^
**1187.1**	**⊕**	asym. str. phosphate,^53,66^ COC,^44^ base CN str., Tyr, Phe^51^
**1223.7**	**⊕**	β-sheet (epithelial cancer),^40^ PO_2_^–^ str., nucleic acids, cellular nucleic acids,^53,67^ a concerted ring mode,^53,56^ collagen^48,53,59,68^
**1253.3**		assym. str. PO_2_^–^,^69^ G, C (NH_2_)^46,53^ aromatic str.,^44,53^ Cys, A, ring str., lipids: def. of CH_2_, CH_3_,^51^ A,^70^ C–N in-plane str.,^53,62^ Cys, T, G,^45^ lipids,^71^ C, T (nucleosome treated by α-chymotrypsin)^50^
**1278.8**	**⊕**	cancer cells, lipid, breast cancer,^44^ collagen^48,53,59^ α-helix (epithelial cancer),^40,53^ CH_2_ (lipids, human colon cancer),^72^ CH_2_ wag.^53,63^ phosphates^53,66^
**1310.5**	**⊕**	C–N asym. str.,^53,62^ ring str. of Phe,^73^ phospholipid (cancer cells),^74^ aromatic amines CH_3_/CH_2_ twist. or bend. of lipid/collagen,^41,53^ CH_3_/CH_2_ twist., wag. and/or bend. of collagens and lipids,^41,53^ C–N str., N–H bend., skeleton str.,^73^ CH_2_ def. twist, Trp, lipids, collagen (cancerous murine fibroblasts)^39^
**1350.5**	**⊕**	keratin-8/18 knock-down cells,^75^ Tyr, T, A, G (nucleosome treated with α-chymotrypsin)^50^
**1393.0**		collagen,^76^ CH rock.,^44,53^ C–N str., in quinoid ring–benzoid ring–quinoid ring,^53,62^ pyrrole in-phase breathing modes (human erythrocytes)^77^
**1394.5**		C–N str., in quinoid ring–benzoid ring–quinoid ring,^53,62^ CH rock.,^44,53^ pyrrole in-phase breathing modes (human erythrocytes)^77^
**1422.0**		G, A, CH def.,^43^ cancerous tissue,^78^ A, G,^70^ A, G (ring breathing modes of the RNA bases),^38,42,53^ NH in-plane def.,^53,56^ O–C=N str.,^79^ A (breast normal and cancer cells and prostate cancer cells),^80^ T, A, G (nucleosome treated with α-chymotrypsin)^50^
**1470.5**		CH def. (nucleosome treated with α-chymotrypsin),^50^ C=N str.,^53,62^ paraffin^53,63^
**1547.1**	**⊕**	C_6_–H def.,^53,56^ NADH,^53,81^ Trp (epithelial cancer)^40,53,82^
**1553.6**		C=C str. Trp,^53,67^ CH_3_, CH_2_ def. (human breast tissue)^61^
**1595.1**		Phe, Tyr,^51^ C=N and C=C str. in quinoid ring (cancerous murine fibroblasts)^39,53,62^
**1872.0**		unassigned
**2129.5**		azide moieties (-N_3_)(breast cancer),^83^ lipid^84,85^

(ii)A431D		
(*ν̃*_obs_)		Assignment (reported *ν̃*_obs_)
**201.4**	**⊕**	unassigned
**262.7**	**⊕**	unassigned
**324.0**	**⊕**	unassigned
**408.8**	**⊕**	phosphatidylinositol^53,57^
**480.2**		C–C–C def.^60^
**513.1**		interchain S–S bridge (human lymphocytes),^54^ Cys S–S str.^44^
**543.2**		S–S str.^44,48,53^
**595.5**		DNA (T, C, G) rings^50^
**672.1**		S=O (lipid),^85^ DNA (T, C, G) rings,^42,45,50,55^ oligomer in solution, possible inclusion of both T and G, G residues associated with furanose rings,^45^ C–S band^86^
**699.5**		methionine trans-C–S str.^44^
**809.7**	**⊕**	O–P–O str. RNA^39,43,53^
**844.8**		C–O–C skeletal mode^44,48,53^
**898.9**	**⊕**	ring breathing mode,^54^ (C–O–C) skeletal mode,^44,48,53^ sym. dioxy str. of the phosphate backbone from nucleic acids and phospholipids^72^
**918.5**		RNA, Pro, hydroxyproline, glycogen, and lactic acid;^39,41,53^ C–C str. of Pro ring/glucose/lactic acid (collagen, human breast tissue, epithelial cancer)^40,53,61,82^
**984.6**		cellulose OCH_3_ polysaccharides^44^
**1139.5**		colorectal cancer cells^86^
**1190.1**		CN str., Tyr, Phe^51^
**1208.6**	**⊕**	hydroxyproline, Tyr (collagen, epithelial cancer),^40,53,61^ Phe,^39,42^ C- C_6_H_5_ str.^41,53,64,67^ A,T (ring breathing modes of RNA),^38,53^ C–C_6_H_5_ str. Trp^40,43,47,53,80,82,87^
**1221.4**		C=N:=C str.^53,62^ T,A^43,53^ =CH bend. (lipids),^43,53^ Amide III, C–N str. and N–H bond coupling^53,88^ asym. str. of PO_2_^–^,^53,67,69^ a concerted ring mode,^53,56^ collagen^48,53,59^
**1239.6**		CH_2_ wag. from Gly Pro,^53,63^ keratin,^58,89^ T, C, A, ring str.,^51^ collagen,^53,63,66^ asym. str. phosphate (PO_5_),^53,82^ C–N str., α-helix conformation (epithelial cancer),^40^ benign tumors (breast cancer),^90^ asym. str. PO_2_®,^41,53^
**1258.7**		benign breast cancer,^44,90^ −CH_2_ rock.,^73^ keratin-8/18 knock-down cells,^75^ C, A, T (ring breathing modes),^38,53^ C, A (epithelial cancer),^40,42^ G, C (NH_2_),^46,53,91^ CH_2_ in-plane def. (lipids),^53,92^ alkyl =C–H cis str. (lipid),^60^ fumarate (breast normal and cancer cells and prostate cancer cells),^80^ epithelial cancer^40^
**1275.2**		CHO rocking,^44,53^ lipids, def. CH_2_, CH_3_,^51^ def. N–H, C–N str.,^44^ lipid, breast cancer^93^
**1336.5**		CH_3_CH_2_ wag. of collagen (epithelial cancer),^53,67,82,94^ CH_2_ def.,^39,53,64^ keratin,^40,53,89^ CH_3_CH_2_ twist,^51,53,64,67^ G,^46,53^ pectin def. CH, ring,^40,44^ Trp.,^89^ G, A,^46,53,91,95^ cellulose def. CH, ring,^44^ polynucleotide chain,^40^ CH_2_ wag. Gly, Pro,^53,63^ A, G ring breathing,^38,53^ C–H def. Trp^41,53^
**1377.1**		CH_3_ sym. def.,^44^ T, A, G,^42,50,51,70,96^ sym. def. CH_3_ (lipid)^53,64^
**1427.4**		CH_2_ bend. and lipids (collagen),^76^ cellulose CH_2_ def.^44^
**1456.7**		CH_2_ str. CH_3_ asym. def.^53,97^ asym CH_3_ bend., CH_2_ sciss. (elastin, collagen, and phospholipids),^53,78,98^ β-carotene, CH_2_ def.^44^ asym. CH_3_ bend., CH_2_ sciss.^78^ CH_2_ def.^53,68^ Keratin-8/18 knock-down cells^75^ CH_2_, CH_3_ sciss., nucleic acid in tissues (benign breast cancer)^53,93,99,100^
**1483.5**	**⊕**	G, A (epithelial cancer) ring breathing modes,^38,40,53^ NH_3_^+^ ^53,97^
**1519.2**	**⊕**	C–C str. (β-carotene),^53,101^ C=C str.,^44^ A, C, G,^51^ C=C str.,^44,53,67^ carotene,^53,81^ −C=C– (carotenoid, epithelial cancer)^40^
**1582.2**	**⊕**	C–C str.,^53,62^ aromatic and aliphatic −C=C–, > N–C=O str.,^44^ G, A,^42^ Phe, Tyr C=C def., Phe,^53,67^ C=C bend.^53,78^ Trp^78^
**2133.0**		azide moieties (N–_3_) (breast cancer)^83^
**2152.0**	**⊕**	CD mode vimentin^102^

(iii)NIH		
(*ν̃*_obs_)		Assignment (reported *ν̃*_obs_)
**394.8**		phosphatidylinositol^53,57^
**455.6**	**⊕**	ring torsion of phenyl,^44,53^ S–S bridge (human lymphocyte)^54^
**513.7**		interchain S–S bridge (human lymphocyte)^54^ S–S str., cysteine^44^
**544.1**		S–S str. C^44,48,53^
**645.1**	**⊕**	unassigned
**674.8**	**⊕**	T, G furanose rings,^45^ C–S str.(colorectal cancer cell)^86^
**723.7**		A (ring breathing mode of RNA bases),^47,53,59^ C–S, C–C^39^
**751.3**	**⊕**	Sym. breathing of Trp,^41,53,67,82^ T,^42,70^ CH_2_,_6_ out-of-plane bend.,^44,53^ nucleic acids, O–P–O sym. str.,^54^ CH_2_ rock., sym. breathing, Trp^95^
**784.5**		DNA,^43,53,103^ sym. phosphodiester str. and ring breathing modes of pyrimidine,^53,56,85^ phosphodiester; C,^46,53^ U, T, C, ring breathing modes backbone O–P–O (785),^38,53^ T, C backbone O–P–O,^96^ C–H str.^44^
**842.5**	**⊕**	glucose (epithelial cancer)^40,53^
**935.3**	**⊕**	C–C skeletal of collagen backbone, α-helix, C–C str. mode of Pro (cancerous murine fibroblasts),^39,41,43,53,61,67,104^ (keratin, epithelial cancer) Val,^40,53,64,89^ R,^42^ C–C backbone (collagen, human breast tissue)^53,61^
**969.6**	**⊕**	lLipids,^53,63^ phosphate monoester groups of phosphorylated,^53,63^ C–O str.,^69^ C–C wag.^53,68^
**995.8**		C–O ribose, C–C,^53,63^ = C–O wag.,^44^ CH_2_ rock.^51^
**1106.5**		C–N str,^42,51^ Phe,^68^ carbohydrates,^63^ CO, CC str. ring,^44^ NH_3_^+^ asym. rock. of His^73^
**1126.1**	**⊕**	C–C str. skeletal of acyl backbone (lipid),^41,53^ C–O ring,^44,48,53^ Pro. (human breast tissue),^61^ C–N, C–C, CO str. skeletal^38,39,43,53,68^
**1156.9**	**⊕**	CC, CN str., CH_3_ rock. (epithelial cancer),^38–40,43,44,51,53,62,82,99^ in-plane vibrations of the conjugated,^53,105^ C=C str.,^53,101^ T, G^50^
**1168.0**	**⊕**	Pro (human breast tissue),^61^ *N* = quinoid ring, = N str. and C–H in-plane bend of Tyr (epithelial cancer) Tyr. (collagen)^40,41,53,62,82^ lipids,^53,63^ C=C str. COH def.,^53,64^ C–C,^53,65^ Phe,^89^ nuclei include the O–P–O str.^69^
**1227.1**	**⊕**	O–P–O asym. str.,^69^ C–O–C str.,^38^ asym. phosphate str.,^53,63^ N–H, C–N str.^53,63^
**1233.8**		O–P–O asym. str.,^69^ a concerted ring mode,^53,56^ keratin,^89^ disordered structure (nonhydrogen bonded)^44^
**1330.6**	**⊕**	keratin-8/18 knock-down cells,^106^ phospholipids,^53,81,98^ collagen,^53,66^ nucleic acids and phosphates,^53^ CH_2_ twist. bend.,^107^ C str. of phenyl and C–C str.^44,53^
**1390.3**		collagen,^76^ CH rock.,^44,53^ C–N str -benzenoid ring, quinoid ring^53,62^
**1430.3**	**⊕**	CH_2_ bend (lipids, collagen),^76^ cellulose CH_2_ def.^44^
**1450.4**	**⊕**	Trp, C–H str., CH_2_, CH_3_, CH_2_CH_3_, methylene def., CH_2_ str., CH_2_,CH_3_ sciss., −CH_2_ bend., CH_3_ bend C–H_2_ twist (collagen, nucleic acid, cytoplasmic region of keratin-8/18 knock-down cells, nucleic acid, tumor cells, lipids, human lymphocyte, cancer cells, aliphatic amino acids, lipids, keratin, vimentin, human breast tissue),^10,39,41,43,44,47,51,53,61–63,68,81,89,102,105,106,108–112^ CH, CH_2_ def. (lipid)^42,96^
**1511.6**		C,^46,53^ A (ring breathing modes, hematopoietic cell),^38,42,53^ C=C str. (lycopene, tetraterpenes)^44^
**1550.1**	**⊕**	C=C str., Trp (epithelial cancer),^40,47,53,67,82^ lipid, oral squamous cell carcinoma^107^
**1583.1**		Phe, Tyr,^51^ C=C def. and C=C bend. of Phe, Trp. and C=C bend (bladder cancer in urine),^53,67,87,113^ Asp, Glu C=O str. (cancerous murine fibroblasts),^39^ C=C olefinic str.,^53,64,97^ cytochrome-c peak (colorectal cancer cell)^86^
**1617.7**		C=C, C–C str., Phe, Tyr., Trp (hematopoietic cell, cancerous murine fibroblasts, epithelial cancer, ricin, and sulfur mustard toxicity in lung cell, porphyrin, NADH)^38–40,42,43,53,62,67,81,82^
**1721.5**	**⊕**	C=O str. (cortisone, citronella, acyclic monoterpenes, eucalyptus)^44,53,57^
**1998.8**		unassigned
**2130.2**		azide moieties (−N_3_), C-D mode (breast cancer, lipid)^83–85^
**2232.5**	**⊕**	-CN str. (breast cancer)^83^
**2322.3**		region of the OH–NH–CH str.^53,114^
**2502.9**		unassigned

a Here, def. = deformation, str. = stretching, sym. = symmetric, asym = asymmetric, wag. = wagging, sciss. = scissoring, and twist. = twisting. The Gauche form is denoted by g, and the Trans form is denoted by t. The abbreviations of amino acids are Tyr: Tyrosine, Leu: Leucine, Gly: Glycine, Ala: Alanine, Met: Methionine, Phe: Phenylalanine, Gln: Glutamine, Lys: Lysine, Arg: Arginine, Asp: Asparagine, Val: Valine, Ile: Isoleucine, Glu: Glutamic acid, His: Histidine, Cys: Cysteine, and Trp: Tryptophan. A: Adenine, G: Guanine, C: Cytosine, and T: Thymine. The major spectral lines observed as major components for Component 2 (*i.e*., components considered to be critical for connecting or conforming outside of the cell frame) are marked with **⊕**. A detailed and complete list of these lines is presented in Table S1, and the difference spectra together with the corresponding list of lines are shown in Figure S1 and Table S3, respectively.

In order to identify the detailed spectral differences among the SERS spectra of the three cell types, a comparative analysis of the spectra was conducted, as shown in Figure 4. We identified seven characteristic regions/peaks (Figure S2 and Table S2): 549–580 cm^–1^ (SS stretching), 596 cm^–1^ (T, C, G ring modes), 651–675 cm^–1^ (S=O, C–S stretching, CO wagging, CC twisting, T, G), 810–845 cm^–1^ (RNA, O–P–O stretching, C–O–C skeletal modes), 1105–1250 cm^–1^ (CN stretching, NH_3_ asymmetric stretching), 1722 cm^–1^ (C=O stretching), and 2131–2153 cm^–1^ (azide moiety, vimentin). These regions were independently analyzed using the peak-fit function of OriginPro 2023b (OriginLab, Northampton, MA, USA), and the spectral intensities were obtained by calculating the integrated areas of the Gaussian-fitted profiles. The resulting spectral intensities for each characteristic region are plotted for A431 (red), A431D (blue), and NIH (green) cells (Figure 5). From the peak trends, only A431 cells exhibited the SS bridge feature, while only A431D cells showed a strong signal corresponding to the T, C, and G ring modes. All three cell types displayed the RNA PO_4_^–^ feature, although NIH cells additionally exhibited CO wagging, CC twisting, and C–O–C skeletal modes, suggesting that NIH cells may support a relatively longer range of molecular networks. The 1722 cm^–1^ (C=O stretching) peak observed in NIH cells cannot yet be concluded to be of specific significance. However, the peak at 2131–2153 cm^–1^ (azide moiety, vimentin), particularly prominent in A431D cells, is consistent with the immunofluorescence results shown in Figure 1.

**Figure 5:**
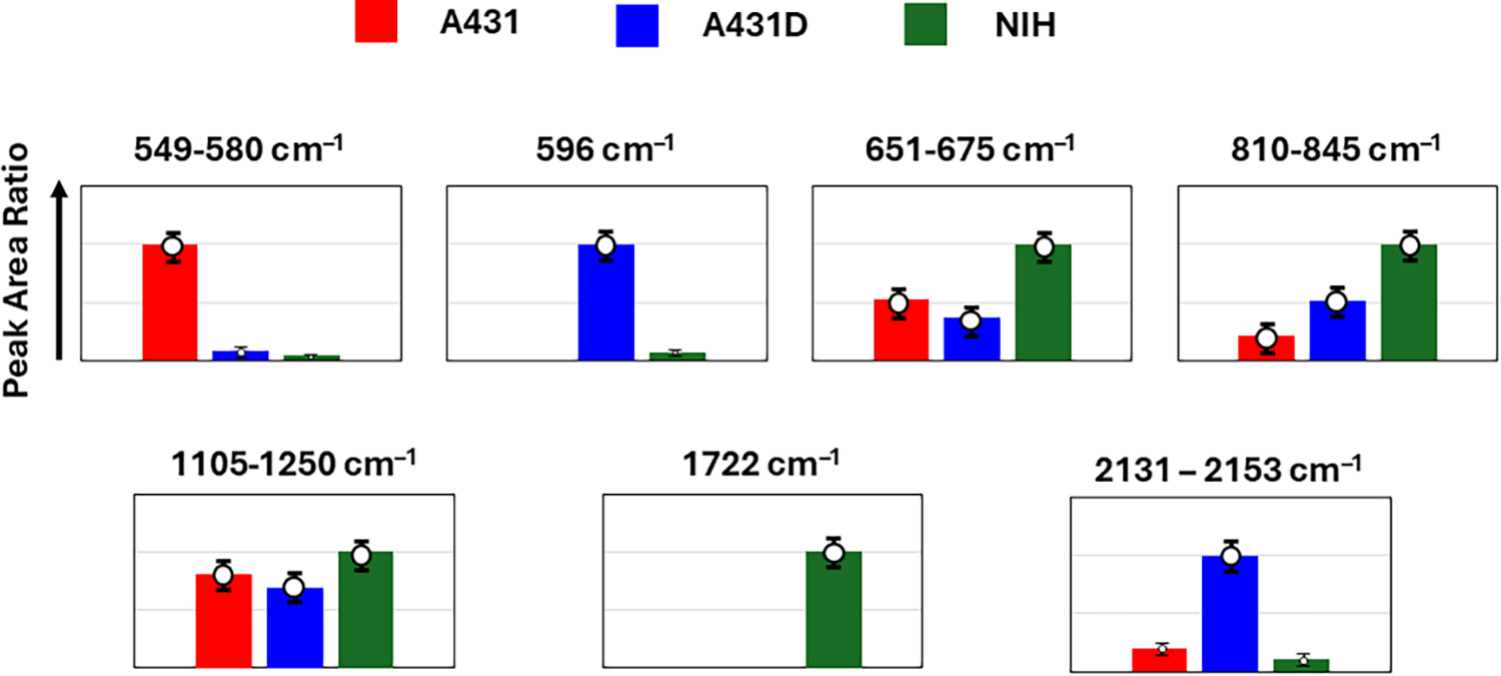
Comparison of normalized peak area ratios (set to 1 for the most intense peak) for selected Raman bands: 549–580 cm^–1^ (SS stretching), 596 cm^–1^ (T, C, G ring modes), 651–675 cm^–1^ (S=O, C–S stretching, CO wagging, CC twisting, T, G), 810–845 cm^–1^ (RNA, O–P–O stretching, C–O–C skeletal modes), 890–1000 cm^–1^ (RNA PO_4_^–^ symmetric stretching, C–O–C skeletal modes), 1105–1250 cm^–1^ (CN stretching, NH_3_ asymmetric stretching), 1722 cm^–1^ (C=O stretching), 1872 cm^–1^ (unassigned), and 2131–2153 cm^–1^ (azide moiety, vimentin) in A431 (red), A431D (blue), and NIH (green) cells.

In order to extract the common components between A431, A431D, and NIH cells, the correlation parameters defined in eq 1 were calculated.

1 $$I_{i,j}\left(\mathrm{\nu ̃}\right)=\left\langle I_{i}\left(\mathrm{\nu ̃}\right)|I_{j}\left(\mathrm{\nu ̃}\right)\right\rangle$$

Here, I_i_(*ν̃*) or *I_j_* (*ν̃*) denotes the SERS signal normalized by the max signal of each spectrum component, *i* or *j* corresponds to either one of A431 cell, A431D cell, and NIH cell at the Raman shift, *ν̃*. The correlated signal map between 250 cm^–1^ and 2800 cm^–1^ is shown in the left column as a three-dimensional plot in Figure 6. A bird’s-eye view of this correlation plot, a contour map, is shown in the center of Figure 6. The SERS spectrum of the diagonal component line is shown in the right column of Figure 6. No distinct differences were observed in the overlaid diagonal spectral components of the correlation contour map. It can be considered that all cells share about the same spectral features. It can be concluded that major spectral features were associated with RNA bases (G, A, C, T) and/or O–P–O- frame stretching as well as motions (*i.e*., str., def., bend., wag., and twist.) of Phe, Tyr, and Trp, and these observed spectral features were almost identical within three cells, as shown in Figure 6b. Thus, the spectral features in the cytoplasm region were regarded as the same between cancerous and normal cells and even between A431 and A431D cells. However, it was noted that, within each cell, component 2 (blue) appeared occasionally within the combined section (purple) (see 3D SERS images in Figure 3), as if they were used as a jointing component to frame the morphology of the cytoplasm region. Detailed analyses are shown in Figure S1 and Table S3. The spectral features observed in multiple cells are marked by [n], where n is the identification group number. Three spectral lines were observed in all three cells: [2] 660 ± 7 cm^–1^: C–S str., C–C twist. Tyr, Phe,^38–44^ A, G, deoxyribose phosphate backbone,^43,45^ [4] 836 ± 5 cm^–1^: phosphodiester,^46^ O–P–O (PO_2_^–^) asym. str., RNA, and Tyr out-of-plane ring breathing, Pro, hydroxyproline,^40–44,47,48^ CH_2_ wag.,^44^ C–H out-of-plane bend. in benzenoid ring,^49^ P, (PO_3_^2–^), PO_4_^2–^ (831),^50^ and [5] 909 ± 7 cm^–1^ **:** amino acids.^51^ It is speculated that several proteins and PO_2_^–^ components of the DNA base must be playing the key role in conforming the cytoplasm region regardless of the cells’ cancerous situation.

**Figure 6:**
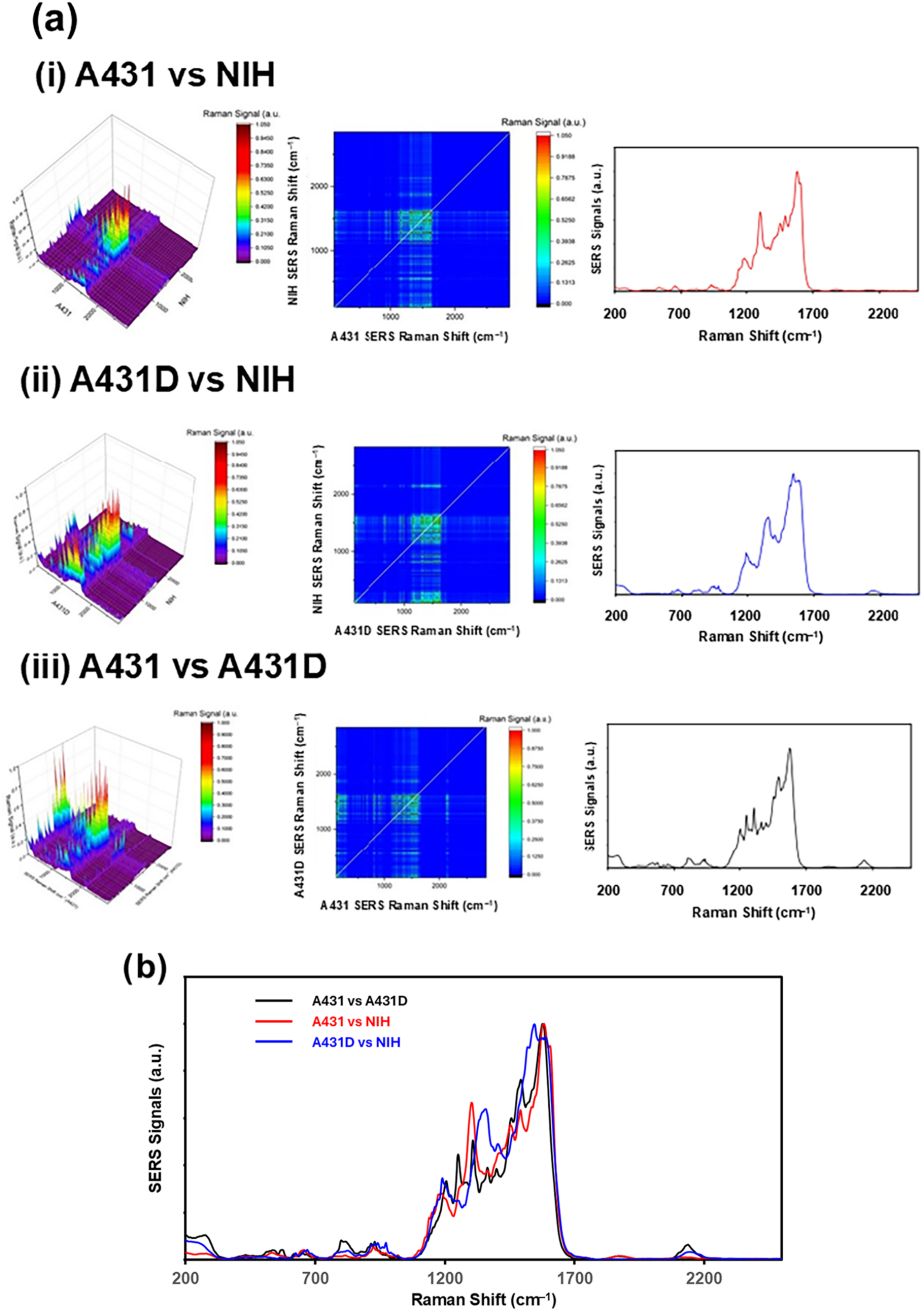
(a) Correlation plot (left), contour map (center), diagonal component of the SERS spectrum (right) between two of three cells (A431 cells, A431D cells, and NIH cells), each defined by eq 1 for (i) A431 vs NIH, (ii) A431D vs NIH, and (iii) A431 vs A431D cells. (b) Overlaid diagonal component of the SERS spectrum.

As a future direction, we can identify earlier time points indicative of changes induced by clobetasol that lead to EMT. Presumably, clobetasol’s effects on the A431 cells begin much earlier than the disappearance of E-cadherin and the appearance of vimentin expression. Comparison of the untreated and treated cells using Raman spectroscopy will allow us to identify EMT in the clobetasol-treated cells much earlier than we are now capable of doing using the detection of E-cadherin and vimentin expression by immunofluorescence microscopy. This information can then be used to identify the earliest molecular changes associated with clobetasol treatment by transcriptome analysis at these initial points of metabolic change associated with EMT in clobetasol-treated A431 cells. Ultimately, this will aid us in dissecting the signaling pathway between clobetasol and loss of E-cadherin/gain of vimentin expression as well as identifying characteristics that enable some cells to escape clobetasol-induced EMT.

## Conclusions

The three-dimensional SERS imaging approach successfully extracted the unique cell conformational changes in cytoskeletal proteins within epithelial vulvar cancer cell line A431, A431D cell (*i.e*., A431 treated with clobetasol), and NIH cells (noncancer cell). A431 cells exhibited bulky components. Although the NIH cells showed a fiber-like conformation, proteins and DNA were localized separately. It was concluded that epithelial–mesenchymal transition is covered by proteins in the conforming cytoplasm.

By tracking protein interactions and surface composition in cells before and after treatment with clobetasol, the distribution of the transition can be reasoned and visualized. Spectral assignments reveal the order of protein gains and losses, while the distinctness of the collected signals from the literature signals provides information about the folding, binding, and repelling forces between these cytoskeletal proteins. On the other hand, three-dimensional Raman imaging of the cytoplasmic region exhibited unique features that may impact the cell construction process. A431D possessed a hybrid component between cancer cells (A431), conforming to a bulky conformation and noncancer cells (NIH) exhibiting a fiber-like conformation. The spectral features were not distinctly utilized to separate the features of each cell status, and the distinct component associated with a particular protein (Tyrosine, Proline, or Phenylalanine) and PO_2_^–^ asymmetric stretching can be speculated to be critical to frame the cell conformation regardless of cancerous status, implying that they will not be impacted by the cancerous status.

While SERS imaging distinguished the cell types, it could not specifically label the spectral component corresponding to keratin or vimentin; further studies could definitively isolate each protein’s contribution. The results presented here revealed distinct 3D morphological features that allowed differentiation between the pretreated (parent) A431 cells and clobetasol-treated A431D cells. A431D cells represent a relatively homogeneous culture of clobetasol-affected cells that express vimentin but have lost E-cadherin expression. However, in culture, the gain of vimentin expression occurs only in a subpopulation of cells rather than uniformly.

Future studies will investigate whether SERS imaging can detect the earliest time points of vimentin upregulation, potentially serving as an early indicator of EMT associated with cancer progression. These observations could be correlated with molecular changes induced by clobetasol treatment by using complementary methods. Moreover, this approach could be extended to systematically investigate cell-to-cell heterogeneity, for example, by combining single-cell proteomics or high-content SERS imaging, to better understand the differential expression of markers such as vimentin and E-cadherin across cell populations.

Additionally, future work could integrate these imaging approaches with proteomic analysis.^52^ Proteomics provides a global view of protein expression, post-translational modifications, and pathway regulation, complementing the high-resolution, single-cell information obtained from our imaging techniques. This combined strategy would strengthen the links between molecular distribution patterns and the underlying proteomic changes in cancer cells, thereby enhancing both the mechanistic and translational effects of this work.

## Materials and Methods

### Cell Culture

A431D cells were generated as described previously.^7^ A431 and A431D cells were cultured in DMEM (Sigma-Aldrich, St. Louis, MO, USA) containing 10% FBS (Sigma-Aldrich, St. Louis, MO, USA) and 1% penicillin–streptomycin (Sigma-Aldrich, St. Louis, MO, USA) at 37 °C in a humidified incubator with 5% CO_2_. NIH3T3 cells were grown in MEM (Sigma-Aldrich, St. Louis, MO, USA) with 10% FBS and 1% penicillin–streptomycin, similar to A431 cells.

### Immunofluorescence Microscopy

Cells (1 × 10^5^ cells/coverslip) cultured on poly-l-lysine-treated coverslips in 12-well plates were fixed with Histochoice MB (VWR, Solon, OH, USA) for 20 min and blocked with 10% Goat Serum (Millipore Sigma, Rockville, MD) in PBS (Kodak, Rochester, NY) for 30 min. Cells were incubated for 1 h with primary antibodies (either rabbit antivimentin; Proteintech, Rosemont, IL), 1:100, mouse anticytokeratin 8 (Abcam, Waltham, MA), 1:100, or mouse anticytokeratin 18 (Abcam, Waltham, MA), 1:100 in 10% GS in PBS. All primary antibodies recognize the respective proteins in both humans and mice. Cells were washed (5 times) with 0.1% PBST and incubated with Alexa 480-conjugated alpaca antimouse secondary antibody or Alexa594-conjugated alpaca antirabbit secondary antibody for 1 h. Cells were washed 5 times with 0.1% PBST. The cells were mounted using VECTASHEILD Antifade Mounting Medium (Vector Laboratories, Newark, CA, USA). Images were acquired using a Zeiss Axiophot microscope with Photometrics Cool Snap HQ2 camera (Photometrics, Tucson, AZ, USA).

### Gold Nanoparticles

A431, A431D, and NIH cells were fixed using the same methods described for immunofluorescence. After fixation, the cells were rinsed with sodium borate buffer (0.1 M, pH 7.4) and incubated for 12 h at 4 °C in sodium borate buffer containing gold nanoparticles with an estimated diameter (d) of 80 ± 1 nm (Ted Pella, Inc., Redding, California, USA). The coverslips were then rinsed with borate buffer, inverted onto glass slides, and sealed with clear nail polish.

### Raman Imaging

Raman spectra were collected by using a WITec Raman alpha300R (Oxford Instruments, WITec, GmbH, Ulm, Germany) confocal Raman imaging system. The microscope can be used for automatic scanning of the specimen by using a computer-controlled sample stage. A laser with a wavelength of λ = 633 nm at ∼10 mW power was used for excitation through a 100× objective (Zeiss, 0.9 NA). Here, spatial resolution was calculated as 0.61× λ/NA ∼ 0.43 μm. The Raman-scattered photons were collected through a photonic fiber and directed to a 300 mm spectrograph equipped with a 600 gr/mm grating and a thermoelectrically cooled CCD detector. To conduct the experiments, a grid consisting of 100 × 100 pixels covering an area of 10 μm × 10 μm was selected over the white-light image, and 10,000 spectra/maps with an integration time of 500 ms/spectrum and a lateral spatial resolution of 0.43 μm were acquired. The typical longitudinal range was 10 μm. Raman spectra in the region of ∼300 to ∼2,800 cm^–1^ were collected. The collected data of the Raman image and the associated Raman shift information were processed using Project Five 5.3 (WITec, Oxford Instruments, GmbH, Ulm, Germany).

## Supplementary Material









## Notes

Documents of Supporting Information for SERS assignments and correlation, Yokoyama–Lewis (PDF)
Animation of three-dimensional SERS imaging of A431 cells (MP4)
Animation of three-dimensional SERS imaging of A431D cells (MP4)
Animation of three-dimensional SERS imaging of NIH cells (MP4)

## Abbreviations

SERS: Surface-Enhanced Raman Scattering
EMT: epithelial–mesenchymal transition
