# Supplementary material for: Visualizing Cytoskeletal Protein Reconstruction of Vulvar Cancer with Surface-Enhanced Raman Spectroscopy and Gold Nanoparticles
Source: ACS Omega. 2026 Jan 16;11(4):5227–40. doi: 10.1021/acsomega.5c07659 (PMC12878780; doi:10.1021/acsomega.5c07659)
Supplement: Supplementary file 1 [file ao5c07659_si_001.pdf]

**Supporting Information**

# Visualizing Cytoskeletal Protein Reconstruction of Vulvar Cancer with Surface-Enhanced Raman Spectroscopy and Gold Nanoparticles

*Kazushige Yokoyama,*[1] Kia Haering,[1] Nicole Mathewson,[2] Patrick Loss,[1] Jani E. Lewis*[2]*

[1]The State University of New York Geneseo College, Department of Chemistry and Biochemistry, Geneseo, NY USA
[2]The State University of New York Geneseo College, Department of Biology, Geneseo NY USA

We attempted to extract the components essential in Component 2 by subtracting the SERS signal (ΔI) between Component 2 ($I_2(\tilde{\nu})$) and Component 1 $I_1(\tilde{\nu})$, *i.e.*, ΔSERS. (**Fig. S1**)

$$\Delta I(\tilde{\nu}) = I_2(\tilde{\nu}) - I_1(\tilde{\nu}) \qquad \textbf{Eq. (S1)}$$

The signals prominent for component 1, (*i.e.,* $\Delta I < 0$) were excluded and those appeared prominently on Component 2 were listed up and the main lines assigned was listed in **Table S3**.

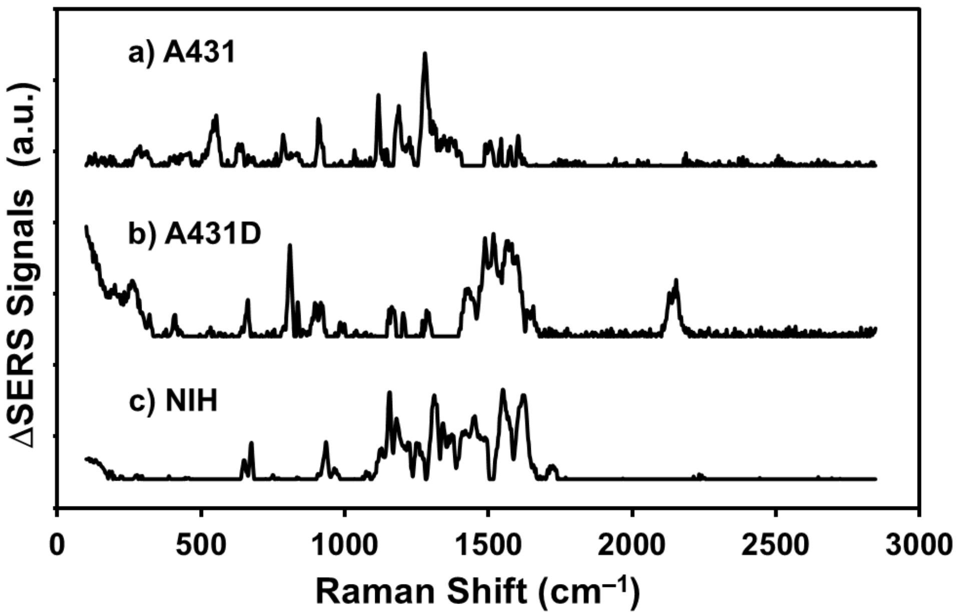


**Figure S1** a)The difference spectrum defined in **Eq. (S1)** of the component which contains more spectral density in the lower wavelength side of the finger point regions (*i.e.,* Amide I, II, and III bands) for a) A431, b) A431D, and c) NIH cells.

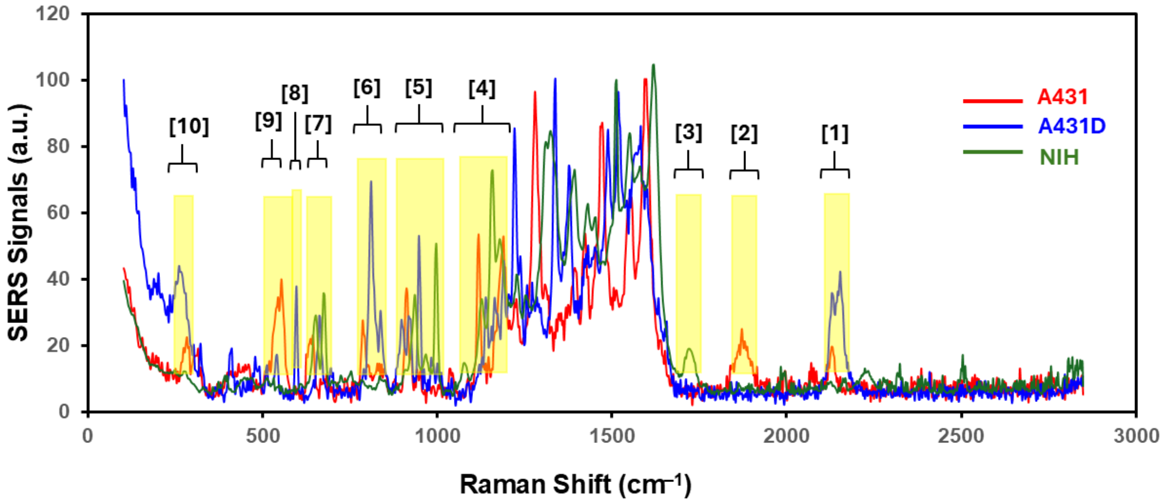


**Figure S2** The two-components combined SERS spectrum of A431, A431D, and NIH cell shown **Fig. 4** are overlayed. The region or peak possess significant spectral intensity change were selected and yellow-highlighted with numbers [1] to [10]. Among those, the spectral intensities were compared for [1], [3]-[4], and [6]-[9] as shown in **Fig. 5**. The [2] and [10] were removed since they were not assigned, and [5] possessed approximately the same intensity profile within three cells.

**Table S1.** The major Raman spectral lines ($\tilde{\nu}_{obs}$) in $cm^{-1}$ picked for (i) A431 Component 1, (ii) A431 Component 2, (iii) A431D Component 1, (iv) A431 Component 2, (v) NIH Component 1, and (vi) NIH Component 2. Here, def. = deformation, str. = stretching, sym. = symmetric, and asym. = asymmetric, wag. = wagging, sciss. = scissoring, and twist. = twisting. Gauche form is given by g, and Trans form is given by t. The abbreviations of amino acids are: Tyr: Tyrosine, Leu: Leucine, Gly: Glycine, Ala: Alanine, Met: Methionine, Phe: Phenylalanine, Gln: Glutamine, Lys: Lysine, Arg: Arginine, Asp: Asparagine, Val: Valine, Ile: Isoleucine, Glu: Glutamic acid, His: Histidine, Cys: Cysteine, and Trp: Tryptophan. A: Adenine, G: Guanine, C: Cytosine, T:Thymine.

## (i) A431 Component 1

| ($\tilde{\nu}_{obs}$) | Assignment (reported $\tilde{\nu}_{obs}$) |
|---|---|
| **642.3** | C-S str., C-C twist.-Tyr(640),[1] crystalline dodecamer, oligomer in both solution and crystalline, possible inclusion of both thymine and guanine, guanine residue associated with furanose rings in the C3' -endo conformation, fibers of DNA of heterogeneous sequence (641),[2] C-C twist. Tyr, Phe (cancerous murine fibroblasts) (642),[3] C-C twist. Tyr[4,5] caffeine,[6] Tyr (thymus chromatin),[7] C-C twist. Tyr (epithelial cancer)[4] (643), Tyr (thymus chromatin)(644)[7] |
| **802.8** | deoxyribomononucleotides (salmon/*Loligo brevis* sperm heads DNA)(800),[8] A form nucleic acids,[2] furanose phosphates backbone (800-815),[2] back bone geometry and phosphate ion interactions (800-1200),[1] C–C str. (lipids/fatty acids)(800-1100),[6] C-H wag. (800-920),[6] uracil-based ring breathing mode (802),[9] p-Cymene delta (ring) (monocyclic monoterpenes)(804),[6] thymol (804)[6] |
| **852.2** | aliphatic CCH def. of Tyr, ring of Tyr (850),[10] Tyr def. str. of CCH, aromatic (cancerous murine fibroblasts)(850),[3] Val and polysaccharides (850),[11] Tyr (850),[6,12] amphetamine (850-950),[13] amino acid side groups (inner histones bound to DNA in chromatin)(851),[14] Tyr, Phe, CC str., ring perturbation of A, $PO_4{}^{2-}$ (nucleosome treated by α-chymotrypsin) histone aromatic side chains (851),[15] (854),[15]Pro, hydroxyproline, Tyr ring breathing, glycogen (852),[5] Tyr, ring breath (hematopoietic cell) (852)[1] (853),[16] Tyr (thymus chromatin) (852, 853),[15] glycogen(carcinogenesis of oesophagus) (852, 853),[12] ring breathing mode of Tyr and C-C str. of Pro ring(853),[4,17] Tyr, aromatic side chains (inner histones bound to DNA in chromatin) (854),[14] (C-O-C) skeletal mode of α-anomers (polysaccharides, pectin) (854),[12] Ring breathing Tyr (854),[18,19] hemiacetal str. and methylene def.(854),[20] pectin (C-O-C) skeletal mode of α-anomers (polysaccharides) (854),[6] ring by Tyr (ricin and sulfur mustard toxicity in lung cell) (854)[19] |
| **879.9** | C-C-N$^+$ sym. str. (lipids), C-O-C ring (ricin and sulfur mustard toxicity in lung cell) (877),[18,19] β-caryophyllene unassigned (sesquiterpenes) (877),[6] C-C-N$^+$ sym. str. (lipids) (877),[19] C-C-N$^+$ str. (breast normal and cancer cells and prostate cancer cells) (878),[21] hydroxyproline, Trp (879),[5] ribose (R17-RNA Virus) (880),[22] Trp, ring def. (880),[6,12] sodium dihydrogen phosphate (amphetamine content) (880)[23] |
| **947.8** | α-helix C-C backbone str., C-O-C str. C-C, protein(oral squamous cell carcinoma) (945),[20,24] ICG (indocyanine green) (945),[25] γ-terpinene $CH_2$ wag., $CH_2$ wag. (monocyclic monoterpenes, marjoram (O. majorana L.)) (947),[6] Amide III' ($D_2O$ solution)( inner histones bound to DNA in chromatin) (950),[14] Val and polysaccharides (950),[11] amphetamine (950-1050),[13,23] C-C (protein, collagen)(tumor cells) (950-1000)[26] |
| **1046.6** | sym. str. of $PO_4^-$ (1044),[5] ribose (R17-RNA Virus) (1045),[22] glycogen, d20 stat hESC-CMs, PC-4 (1048),[27] glycogen (carcinogenesis of oesophagus) (1048) [12,16] |
| **1167.2** | C–C str. (crocetin) (tetraterpenes, stigma) (1165),[6] Pro (human breast tissue) (1166),[28] N= str. quinoid ring, ==N str. and C-H in plane bend. (1167),[29] lipids(1168),[30] C=C str. def. COH (lipid) (1168),[23] C-C str., carotenoid(1168),[31] Tyr (collagen) (1168)[5] |
| **1253.3** | assym. str. $PO_2^-$(melanocytes) (1251-1257),[32] Amide III (DNA in chromatin) (1252),[14] G, cytosine ($NH_2$) (1252)[18,33] aromatic str. (1252),[6,18] Amide III (human lymphocyte) (1240-1252 $cm^{-1}$),[34] Amide III (thymus chromatin) (1252, 1253),[15] thymine, Cys, A, ring str. (IRPT/J791), lipids: def. of $CH_2$, $CH_3$ (1254),[10] Amide III, A (CHL-cell metaphase chromosomes) (1254),[35] C-N in plane str. (1254),[18,29] Cys and T (1255),[2] Cys and G containing oligomers and polymers (1255),[2] lipids (1255),[36] Amid III, C+T (nucleosome treated by α-chymotrypsin) (1255)[15] |
| **1394.5** | C-N str., in quinoid ring-benzoid ring-quinoid ring (1392),[18,29] CH rock. (1393),[6,18] DA-HEK293 human (1393),[37] pyrrole in-phase breathing modes (human erythrocytes) (1396)[38] |
| **1422.0** | deoxyribose, (B,Z-marker) (1420–1480),[18,33] G, A, CH def. (1420–1480),[19] cancerous tissue of the nasopharynx (1420-1480),[39] A, G (CHL-cell metaphase chromosomes) (1421),[35] deoxyribose, (B,Z-marker) (1421),[18,33] A, G (ring breathing modes of the DNA/RNA bases, hematopoietic cell) (1421),[1,18] deoxyribose, (B,Z-marker) (1422, 1424),[18,33] NH in-plane def.(1423),[9,18] O-C=N str.(1423),[40] A (breast normal and cancer cells and prostate cancer cells) (1423),[21] A, G (thymus chromatin) (1423),[7] deoxyribose, (B,Z-marker) (1424),[18,33] T, A, G (nucleosome treated by α-chymotrypsin) (1424)[15] |
| **1470.5** | CH def. (nucleosome treated by α-chymotrypsin)(1468),[15] C=N str. (1470),[18,29] paraffin (1472)[18,30] |
| **1553.6** | C=C str. Trp, porphyrin (1552),[18,41] Amide II (human lymphocyte) (1554),[34,42,43] chlorophyll a 5 coordinated (chlorophylls) (1554, 1555),[6] $CH_3$, $CH_2$ def. (human breast tissue) (1554)[28] |
| **1595.1** | Phe, Tyr (IRPT/J810)(1593),[10] C=N and C=C str. in quinoid ring(1593),[18,29] C=N and C=C str.(cancerous murine fibroblasts) (1595)[3], DC473 (colorectal cancer cell) (1597)[44] |
| **1872.0** | Unassigned |
| **1997.0** | Unassigned |
| **2077.2** | Unassigned |
| **2129.5** | azide moieties ($-N_3$)( Breast cancer) (2120),[45]C-D str. (D7-Glc & CD-AA) (lipid) (2133)[46,47] |

(ii) A431 Component 2

| $(\tilde{\nu}_{obs})$ | Assignment (reported $\tilde{\nu}_{obs}$) |
|---|---|
| **279.5** | unassigned |
| **312.9** | unassigned |
| **406.3** | unassigned |
| **456.3** | ring torsion of phenyl-2 (454),[6,18] S-S bridge (human lymphocyte) (458-567)[34] |
| **549.8** | cholesterol(548),[18,48]SS str. (DNA in chromatin) (550),[14] CL domain S-S bridge(human lymphocyte) (551)[34] |
| **637.2** | ribose (S-S, C-S bond) (R17-RNA virus)(635),[22] C-S str., C-C twist.-Tyr (640) (hematopoietic cell)[1,18] |
| **683.2** | G, B- and disordered DNA (salmon/*Loligo brevis* sperm heads DNA) (681),[8] G (CHL-cell metaphase chromosomes) (681),[35] G (DNA in chromatin) (681),[14] G (thymus chromatin) (682),[7] T, G, base interactions of B-form DNA (nucleosome treated by $\alpha$-chymotrypsin) (682),[15] G (duplex of the dodecamer, d(CGCGAATTCGCG)) (683),[2] C2'-endo anti configuration involving G residue of the oligomer (683)[2] |
| **788.1** | phosphate furanose backbone (786),[2] crystalline dodecamer (786),[2] d(CGCGAATTCGCG) (786),[2] DNA: O-P-O, cytosine, uracil, thymine (786),[4,18] pyrimidine ring breathing mode (786),[9,18] DNA: O-P-O, C, U, T (epithelial cancer) (786),[4] C, uracil (R17-RNA virus) (787),[22] nucleic acids (787),[18,29] phosphatidylserine (787),[18,48] C, U, T(microbial cells) (787),[29] $\alpha$-pinene C–H wag. (bicyclic monoterpenes) (787),[6] C, T, P (nucleosome treated by $\alpha$-chymotrypsin) (787),[15] DNA: O-P-O, C, T (CHL-cell metaphase chromosomes) (788),[35] dodecamer in crystalline, backbone and base contribution (788-790),[8] C'5-O -P-O-C'3 phosphodiester bands in DNA (788),[18,19] O-P-O str. DNA(788),[18,19] C, T, P (thymus chromatin) (788),[7] O-P-O str. DNA (ricin and sulfur mustard toxicity in lung cell) (788),[19] thymine, deoxyribose phosphate backbone (O-P-O), d(CGCGAATTCGCG) (790)[2] |
| **843.4** | glucose (epithelial cancer) (842),[4,18] 1,8-cineol C-H wag. (bicyclic monoterpenes) (843)[6] |
| **911.2** | DA-hDAT-HEK293 (dopamine transporter in cell) (911),[37] glucose(913)[18,48] |
| **1040.2** | normal and tumor tissues (1040, 1041),[18,49] HEK293 (dopamine transporter in cell) (1041)[37] |
| **1117.8** | $CH_{2,6}$ in-plane bend and Cl-$C_\alpha$-$H_\alpha$ bend (1115, 1116),[6,18] alkyl C-C trans and gauche str. (lipid) (1116),[20] Glucose (1117),[18,48] C-C str. (breast lipid) (epithelial cancer) (1117-1119 $cm^{-1}$),[4,18] CN str. (IRPT/J783) (1118),[10] Pro (human breast tissue) (1118, 1119),[28] C-C str. (breast lipid) (epithelial cancer) (1119)[4] |
| **1187.1** | asym. str. phosphate (1185-1300),[18,50] COC (1185),[6] DA-HEK293 (dopamine transporter in cell) (1186),[37] polysaccharides (MCF10A/MDA-MB-435) (1187),[51] nucleotides: base CN str., Tyr, Phe (IRPT/J786) (1188)[10] |
| **1223.7** | β-sheet (epithelial cancer) (1221),[4] $PO_2^-$ asym. str. (B-DNA in control nuclei melanocytes) (1222),[32] $PO_2^-$ str., nucleic acids, cellular nucleic acids (1223),[18,41] a concerted ring mode (1223),[9,18] collagen (carcinogenesis of oesophagus) (1223),[12,16,42,52] nuclei isolated from cell, irradiated nuclei, melanocytes(1224, 1225-1242)[32] |
| **1278.8** | cancer cells, lipid, breast cancer(1277),[6] thymus chromatin (1277),[7] collagen, (carcinogenesis of oesophagus) (1278),[12,16,18] $\alpha$-helix (epithelial cancer) (1279),[4,18] $CH_2$ (lipids, human colon cancer) (1280),[53] $CH_2$ wag. from Gly backbone & proline side chains(1280),[18,30] collagen, nucleic acids and phosphates (1280)[18,50] |
| **1310.5** | C-N asym. str. (1308),[18,29] ring str. of Phe (dopamine transporter in cell) (1308-1310),[37] phospholipid (cancer cell - U87-MG glioblastoma cells) (1308),[54] aromatic amines $CH_3$/$CH_2$ twist. or bend. of lipid/collagen (1308),[5,18] $CH_3$/$CH_2$ twist., wag. and/or bend. of collagens and lipids (1309),[5,18] Amide III C-N str., N-H bend., skeleton str., DA-hDAT-HEK293, (dopamine transporter in cell) (1311-1312),[37] $CH_2$ def. twist, Trp, lipids, collagen (cancerous murine fibroblasts) (1312)[3] |
| **1350.5** | DA (dopamine transporter in cell) (1348),[37] keratin-8/18 knock-down cells (1350),[55] carbon particle (1350),[18,56] core histone Tyr, T, A, G (nucleosome treated by $\alpha$-chymotrypsin) (1353)[15] |
| **1393.0** | DA (dopamine transporter in cell) (1390),[37] Amide III (collagen) (1390),[57] CH rock. (1391),[6,18] C-N str., in quinoid ring-benzoid ring-quinoid ring (1392),[18,29] CH rock. (1393),[6,18] DA-HEK293 (dopamine transporter in cell) (1393),[37] pyrrole in-phase breathing modes (human erythrocytes) (1396)[38] |
| **1474.6** | paraffin (1472),[18,30] lipids, $CH_2$, $CH_3$ def. (IRPT/J802) (1474),[10] DA-HEK293 (dopamine transporter in cell) (1474)[37] |
| **1480.4** | Trp, Amide II (MCF10A/MDA-MB-435)(1479),[51] poly ethylene glycol 600 (1479),[58] G, A (R17-virus) (1480),[22] Amide II CN str. & in-plane bend. of the N-H group (1480-1575 $cm^{-1}$),[18,30] Amide II, N-H def., C-N str. (1480-1543 $cm^{-1}$),[6] G, A (R17-RNA virus) (1482)[22] |
| **1510.7** | C (1508),[18,33] A (CHL-cell metaphase chromosomes) (1510),[35] C (1510),[18,33] ring breathing modes A, DNA/RNA, (hematopoietic cell) (1510),[1,18] lycopene C=C str. tetraterpenes, tomato (lycopersicon esculentum Mill.) (1510),[6] C=C bonds (1510),[6] A (thymus chromatin) (1512)[7] |
| **1547.1** | $C_6$-H def. (1545),[9,18] chlorophylls (1545, 1549),[6] Amide II (melanocytes) (1545),[32] NADH (1546),[18,59] heme aggregation, heme Fe atom in a low - spin state (S=0) (human erythrocytes) (1546),[38] Amide II (IRPT/J807) (1548),[10] Trp (epithelial cancer) (1548),[4,17,18] Amide II (DNA repair, melanocytes) (1549)[32] |
| **1597.1** | C=N, C=C str. (cancerous murine fibroblasts) (1595),[3] DC473 phenyl (colorectal cancer cell) (1597),[44] (R17-virus) (1600),[22] Amide I C=O str. peptide backbone hydrogen bonding of peptide group (1600-1800),[1,18,36] Amide I (1600-1700)[20] human tissue (bladder cancer in urine) (1600),[60] DA (dopamine transporter in cell) (1600),[37] aryl ring (lignin, lipid) (1600),[47] C=C str. (1600),[6] ring str. (1600-1650),[6] harpagoside C=O, -C=C- and benzene ring str. (1600-1700),[6] aryl ring (Lignin) lipids (1600),[47] Amide I band of nucleic acids (melanocytes) (1600-1680)[32] |

(iii) A431D Component 1

| $(\tilde{\nu}_{obs})$ | Assignment (reported $\tilde{\nu}_{obs}$) |
|---|---|
| **340.4** | ribose, cytosine(R17-virus)(350)[22] |
| **367.8** | cytosine (R17-virus) (385)[22] |
| **480.2** | C-C-C def. (479),[20] DNA(481),[16,18] glycogen (carcinogenesis of esophagus) (481)[12] |
| **513.1** | Interchain S-S bridge (human lymphocyte) (510),[34] Cys S–S str. g–g–g cell-AF-1(512)[6] |
| **543.2** | S-S str, g-g-t, Cys, t-g-t (540),[6,12,18] glucose-saccharide band (540),[18,48] anthocyanidin mono glycosides (540)[6] |
| **595.5** | phosphatidylinositol(596),[18,48] DNA (T, C, G) rings, A-form DNA, intermediate B-form (nucleosome treated by $\alpha$-chymotrypsin) (597)[15] |
| **672.1** | guanine (R17-RNA virus) (670),[22] T, G (CHL-cell metaphase chromosomes) (670),[35] S=O (DMSO) (lipid) (670),[47] DNA (T, C, G) rings, base interactions of A-, B-form DNA (nucleosome treated by $\alpha$-chymotrypsin) (670),[15] T (inner histones bound to DNA in chromatin) (671),[14] T (thymus chromatin) (671),[7] T, G (duplex of the dodecamer, d(CGCGAATTCGCG)) (672),[2] oligomer in solution, possible inclusion of both T and G, G residue associated with furanose rings in the C3' -endo conformation (672),[2] C-S str. (colorectal cancer cell) (674)[44] |
| **699.5** | Phosphatidylcholine, lipids (breast, normal and breast/prostate cancer cells) (699),[21] methionine trans-C–S str. (700-745)[6] (700-745),[12,18] Cholesterol (702)[18,48] |
| **724.2** | A (R17-virus) (722),[22] DNA (carcinogenesis of oesophagus) (722),[12,16,18] C-S, C-C (cancerous murine fibroblasts) (724),[3] A, ring breathing mode of DNA/RNA bases, (hematopoietic cell) (725),[1,18] A (epithelial cancer) C-S str., $CH_2$ rock., A (726)[4,18] |
| **770.8** | DNA 5',3'-deoxyribosephosphate backbone (salmon*/Loligo brevis* sperm heads DNA)(769),[8] $\beta$-caryophyllene unassigned (sesquiterpenes) (769),[6] DA (dopamine transporter in cell) (772)[37] |
| **826.8** | C2'- endo conformations (825-840),[2] phosphodiester (825),[18,33] Unassigned, salmon*/Loligo brevis* sperm heads DNA (826),[8] O-P-O str. (epithelial cancer) DNA, phosphodiester, Pro, hydroxyproline, out of plane ring breathing, Tyr (cancerous murine fibroblasts) (826, 828),[3,4,33,42] Pro, Tyr: def. (CCH) aliphatic, Tyr (ring) (IRPT/J774) (827),[10] Pro, hydroxyproline, Tyr, $PO_2^-$ str. of nucleic acids (827),[5,18] aromatic side chains (inner histones bound to DNA in chromatin) (828),[14] C2'-endo furanose population of the dodecamer in the crystal and solution states (828-833),[2] out-of-plane ring breathing, Tyr/O-P-O str. DNA (828),[4,17,18] phosphodiester (828),[19,33,42] ring breathing Tyr (828),[18,19] $\alpha$-bisabolol $CH_2$ wag. (sesquiterpenes) (828),[6] Tyr (thymus chromatin) (828),[7] O-P-O asym. str. (828), ring breathing Tyr (ricin and sulfur mustard toxicity in lung cell) (828)[19] |
| **844.8** | glucose (epithelial cancer) (842),[4,18] 1,8-cineol C-H wag. bicyclic monoterpenes (843),[6] monosaccharides (α-glucose), (C-O-C) skeletal mode disaccharide (maltose) (847)[6,12,18] |
| **948.0** | $\alpha$-helix C-C backbone str., C-O-C str. (945),[20] C-C (oral squamous cell carcinoma) (945),[24] indocyanine green (945),[24] $\gamma$-Terpinene $CH_2$ wag. (947),[6] $CH_2$ wag., Pro, Val, polysaccharides (950),[11,18] amphetamine (950–1000),[13,23,42] C-C, collagen (tumor cells) (950–1000)[26] |
| **984.6** | 1,8-cineol $CH_2$ wag., bicyclic monoterpenes (984),[6] cellulose $OCH_3$ polysaccharides (985),[6] polysaccharides (MCF10A/MDA-MB-435) (986)[51] |
| **1012.0** | C-O-H def. (dopamine transporter) (1010-1012),[37] (1014),[37] poly ethylene glycol-600 (1013)[58] |
| **1088.8** | C-C str. g (1086),[18,52] $CO_3^-$, $PO_4^-$, C-C skeletal of acyl back-bone in lipid (gauche) (1087),[5,18] C-C str. (1087-1090)[4,18] $PO_2^-$ str. (1087-1090 )[4,18] C-C str. $PO_2^-$ str. (epithelial cancer) (1087, 1090),[4] CC str. (lipid),. CC skeletal, CN str. (cancerous murine fibroblasts) (1088),[3] $O_2^-$ sym. str. (melanocytes) (1088),[32] sym. phosphate str. (1090),[18,30] DA-hDAT-HEK293 (dopamine transporter) (1090),[37] sym. str. phosphate backbone (lipid) (1090-1100)[47] |
| **1139.5** | hDAT-HEK293 (dopamine transporter) (1138, 1140),[37] DC473 Phenyl(colorectal cancer cell) (1141)[44] |
| **1190.1** | nucleotides base, CN str., Tyr, Phe (IRPT/J786) (1188),[10] hDAT-HEK293 (dopamine transporter in Cell) (1190)[37] |
| **1221.4** | C=:N=C str. (1220),[18,29] ß-sheet (epithelial cancer) (1220-1221),[4,18] T,A (DNA/RNA) (1220-1284),[18,19] Amide III (1220-1284),[18,19] ==CH bend. (lipids) (1220-1284),[18,19] Amide III, C-N str. & N-H bond coupling (1220-1300),[11,18] T, A (ricin and sulfur mustard toxicity in lung cell) (1220-1284),[19] asym. str. of $PO_2^-$, B-DNA (melanocytes) (1222),[32] str. of $PO_2^-$, cellular nucleic acids (1223),[18,41] A concerted ring mode (1223),[9,18] collagen (carcinogenesis of oesophagus) (1223)[12,16,18] |
| **1239.6** | $CH_2$ wag. from Gly Pro (1237),[18,30] C, U (R17-RNA virus) (1238),[22] Amide III (1239),[18,43] keratin (MCF10A/MDA-MB-435) (1237),[27] (1240),[51] T, C, A, ring str. (IRPT/J790) (1240),[10] histones bound to DNA in chromatin (1240),[14] RNA (hematopoietic cell) (1240),[1,18] collagen (1240),[30,42,50] asym. str. phosphate ($PO_5$) (1240),[17,18] poly ethylene glycol (1240),[58] C-N str., α-helix conformation (epithelial cancer) (1240),[4] benign tumors (breast Cancer) (1240),[61] nucleosome treated by $\alpha$-chymotrypsin (1240),[15] core histone (1240),[15] asym. str. $PO_2^-$(1241),[5,18] HEK293 (1241) |
| **1258.74** | benign breast cancer (1256),[6] -$CH_2$ rock. (dopamine transporter) (1256),[37] keratin-8/18 knock-down cells (1257),[55] cytosine (duplex of the dodecamer, d(CGCGAATTCGCG)) (1257),[8] A, T (ring breathing modes of the DNA/RNA bases) (1257),[1,18] A, T, ring breathing modes of the DNA/RNA, protein (hematopoietic cell) (1257),[1] thymus chromatin (1257),[7] A (1258),[4,18] C A (thymus chromatin) (1258),[7] A, C (epithelial cancer) (1258),[4] G, C ($NH_2$) (1259),[18,33] fatty acids (MCF10A/MDA-MB-435) (1260),[51] tumors (1260),[18,62] $CH_2$ in-plane def. (lipids) (1260),[18,63] alkyl =C-H cis str. (Lipid) (1260),[20] fumarate (breast normal and cancer cells and prostate cancer cells) (1260),[21] epithelial cancer (1260),[4] benign and malignant tissues (breast cancer) (1260) [61] |
| **1275.2** | CHO rocking (1273),[6,18] lipids, def. $CH_2$, $CH_3$ (IRPT/J793) (1274),[10] inner histones bound to DNA in chromatin (1274),[14] N-H def., C-N str., (1275),[6] lipid, breast cancer (1277),[64] thymus chromatin (1277)[7] |

| | |
|---|---|
| **1336.5** | collagen, lipid phenotype of cancer associated fibroblast: $CH_3CH_2$ wag.(1334),[65] :$CH_2$ def. (1335),[3] Keratin (MCF10A/MDA-MB-435) (1335),[51] $CH_3CH_2$ wag. def. (collagen) polynucleotide chain (DNA purine bases) (1335),[17,18,41] (1335-1345),[4,18] $CH_3CH_2$ twist wag. (nucleic acid) (1335),[18,41] G (1335),[18,33] pectin def. CH, ring (polysaccharides) (1335),[6] $CH_3CH_2$ wag. of collagen (epithelial cancer) (1335),[4] Trp. MCF10A/MDA-MB-435 (1336),[51] G, A duplex of the dodecamer, d(CGCGAATTCGCG) (1336),[8,33,42] Polynucleotide chain (DNA purine bases) (1336),[4,18] def. $CH_3$, $CH_2$ twist., collagen (1336),[18,66] cellulose def. CH, ring (polysaccharides) (1336),[6] polynucleotide chain (DNA-purine bases) (epithelial cancer) (1336),[4] polysaccharides (MCF10A/MDA-MB-435) (1337),[51] $CH_2$ wag. Gly, Pro (1337),[18,30] A, G ring breathing (1337),[1,18] C-H def. Trp (1337),[5,18] A, G, ring breathing DNA/RNA, C-H def., (hematopoietic cell) (1337),[1] $CH_2$, $CH_3$ twist. wag., (IRPT/J796) (1338),[10] $CH_2$ def., A and G of DNA/RNA, Trp, (lipid, glioblastoma multiforme)(1338)[67] |
| **1377.1** | $CH_3$ sym. def. ($\alpha$-bisabolol, polysaccharides) (1375),[6] T, A, G (treated by $\alpha$-chymotrypsin) (1375),[15] T, G, A (nucleus-cytoplasm, CHO cells) (1375),[68] T, A, G (thymus chromatin) (1376),[7] A, G, (CHL-cell metaphase chromosomes) (1377),[35] paraffin (1378),[18,30] sym-$CH_3$ def. (lipid) (1379)[18,66] |
| **1456.7** | $CH_2$ str. $CH_3$ asym. def. (1454),[18,43] asym. $CH_3$ bend., $CH_2$ sciss. (elastin, collagen, & phospholipids) (1454),[39,42,62] $\beta$-carotene, $CH_2$ def. (tetraterpenes, cucurbita pepo L.) (1454),[6] asym. $CH_3$ bend., $CH_2$ sciss. (nasopharynx cancerous tissue) (1454),[39] deoxyribose (1455),[18,33] $CH_2$ def. (1455),[18,52] keratin-8/18 knock-down cells (1457),[55] deoxyribose (1457),[18,33] $CH_2$,$CH_3$ sciss., nucleic acid in tissues (benign breast cancer)(1458)[42,64,69,70] |
| **1483.5** | G, A (R17-RNA virus) (1482),[22] G, A ring breathing modes (hematopoietic cell) (1485),[1,4,18] $NH_3^+$ (1485-1550),[18,43] G, A (epithelial cancer) (1485)[4] |
| **1519.2** | C-C str. ($\beta$ -carotene) (1517),[18,71] C=C str. (capsanthin) (1517),[6] A, C, G (IRPT/J804) (1518),[10] C=C str., porphyrin (1518),[18,41] C-C, C=C str. (1518),[18,41] cis-bixin, C=C str. (tetraterpenes, annato, bixa orellana L.) (1518),[6] carotene (1520),[18,59] -C=C- carotenoid (1520-1538),[4,18] -C=C- (carotenoid, epithelial cancer) (1520)[4] |
| **1541.1** | carbonyl, aromatic (1540-1680),[18,72] human RBC (1543)[18,29] |
| **1582.2** | C-C str. (1580),[18,29] aromatic and aliphatic -C=C-, >N-C=O str. (1580-1635),[6] G, A (thymus chromatin) (1580),[7] MBA ring breathing mode (4-mercaptobenzoic acid in cells) (1580),[73] heme aggregation, Fe atom in the high-spin (S=2, 0) human erythrocytes (1581, 1582),[38] Phe, Tyr (IRPT/J809) (1582),[10] C==C def., Phe (1582),[18,41] C=C bend. Phe (1583),[18,39] Trp and C=C bend. (nasopharynx cancerous tissue) (1583),[39] malignant tissue (bladder cancer in U) (1584)[60] |
| **1627.6** | C=C str. (nucleotides, lipids, olefinic, IRPT/J812) (1625),[10] C=O str. (1625),[40] $C_\alpha$=$C_\alpha$ str. (1628),[6,18] C=O str. ($\beta$-form polypeptide) (1628)[18,74] |

(iv) A431D Component 2

| ($\tilde{\nu}_{obs}$) | Assignment (reported $\tilde{\nu}_{obs}$) |
|---|---|
| **201.4** | unassigned |
| **262.7** | unassigned |
| **324.0** | unassigned |
| **408.8** | Phosphatidylinositol (415)[18,48] |
| **542.0** | Cys, S-S str. g-g-t, t-g-t (540),[6,12,18] glucose-saccharide band (540),[18,48]anthocyanidin monoglycosides (540)[6] |
| **662.5** | C-S str. cystine (collagen) (662)[5,17,18] |
| **809.7** | C3'-endo deoxyribose phosphate backbone (d(CGCGAATTCGCG)) (807),[2] DA-hDAT-HEK293 (dopamine transporter) (807),[37] O-P-O str. RNA (cancerous murine fibroblasts) (809),[3] sym. dioxy str. phosphate backbone (nucleic acids and phospholipids, human colon cancer) (810),[53] phosphodiester (810),[18,33] O-P-O str. ((ricin and sulfur mustard toxicity in lung cell)) (811),[18,19] carvacol (811)[6] |
| **834.8** | DNA: backbone (CHL-cell metaphase chromosomes) (832),[35] diester -O-P-O- str. (histones bound to DNA in chromatin) (832),[14] Tyr (thymus chromatin) (832),[7] d(CGCGAATTCGCG) (833, 834),[2] pdT (833),[2] phosphate furanose backbone (833),[2] C2'-endo furanose, furanose phosphate (833, 835),[2] Tyr, exposed/buried (human lymphocyte) (835)[34] |
| **857.5** | Pro, Tyr, C-C str., CCH def. ring breathing, Tyr (polysaccharide) (855),[18,41] CCH def. Phe, olefinic (polysaccharide) (855),[18,66] Tyr (human lymphocyte) (855),[34] DA-hDAT-HEK293 (dopamine transporter) (855),[37] C-C str., Pro (human breast tissue) (855),[28] Pro (collagen) (855),[57] Pro, hydroxyproline, Tyr (856),[5,18]C-C str., Pro (collagen) (856),[18,28] C-C str. Pro and hydroxyproline (collagen) (856),[5,18] C-C str., Pro (human breast tissue) (856),[28] Tyr (collagen) (859),[18] DA-HEK293 (dopamine transporter) (859),[37] Tyr (collagen, epithelial cancer) (859)[4] |
| **898.9** | phosphodiester, deoxyribose (896),[18,33] RP (thymus chromatin) (896),[7] ring breathing mode (human lymphocyte) (897),[34] monosaccharides ($\beta$-glucose), (C-O-C) skeletal mode, (disaccharide, maltose) (898),[6,12,18] DA-HEK293(dopamine transporter) (898),[37] ribose-phosphate; saccharides (IRPT/J776) (899),[10] sym. dioxy str. of the phosphate backbone from nucleic acids and phospholipids (human colon cancer) (900),[53] -C-O-C- str. of morphine in the poppy (900-1100)[6] |
| **918.5** | ribose, RNA, Pro, hydroxyproline, glycogen and lactic acid (cancerous murine fibroblasts) (917, 918),[3,5,18] HEK293(dopamine transporter) (917),[37] glycogen and lactic acid (carcinogenesis of oesophagus) (918),[12,16,42] C-C str. of Pro ring/glucose/lactic acid (collagen, human breast tissue, epithelial cancer) (920)[4,17,18,28] |
| **984.7** | 1,8-cineol $CH_2$ wag. (bicyclic monoterpenes) (984),[6] cellulose $OCH_3$ (polysaccharides) (985),[6] polysaccharides (MCF10A/MDA-MB-435) (986)[51] |

| | |
|---|---|
| **1000.6** | IRPT/J779 (998),[10] CC str. (human RBC) (999),[18,75] sym. dioxy str. of the phosphate backbone from nucleic acids and phospholipids (human colon cancer) (1000),[53] Phe, Bound & free NADH (1000),[18,59] out-of-phase C-C-O str. (1000-1075),[6] in-plane rock. $CH_3$ groups attached to the polyene chain and coupled with C-C bonds (1000-1020),[6] Phe (MCF10A/MDA-MB-435) (1001),[51] A, U, C(R17-RNA virus) (1001),[22] Phe aromatic ring (cancerous murine fibroblasts, lipids) (1001),[3] sym. ring breathing mode of Phe (epithelial cancer) (1001),[4,17,18] C-C aromatic ring str.(1002),[18,43] Phe (1002),[5,18,59] Phe (collagen assignment, human breast tissue) (1002) [18,28] |
| **1043.7** | normal and tumor tissues (1041),[18,49] HEK293 (dopamine transporter) (1041),[37] carbohydrates (1043),[18,30] Pro (collagen) (1043),[18,28] Pro (human breast tissue) (1043),[28] sym. str. of $PO_4^-$(1044),[5,18] ribose (R17-RNA virus) (1045)[22] |
| **1166.6** | hDAT-HEK293 (dopamine transporter) (1164),[37] C–C str.(crocetin, tetraterpenes, stigma) (1165),[6] Pro (human breast tissue) (1166, 1167),[28] N= quinoid ring, ==N stretching and C-H in plane bend. (1167),[18,29] lipids (1168),[18,30] C=C str. COH def. (lipid) (1168),[18,23] C-C str. carotenoid (1168)[18,31] |
| **1208.6** | hydroxyproline, Tyr (collagen, epithelial cancer) (1206),[4,18,28] Tyr (human breast tissue) cell-AO (1206)[28] polysaccharides (MCF10A/MDA-MB-435) (1207),[51] Tyr, Phe (thymus chromatin) (1207, 1210),[7] Tyr, Phe (cancerous murine fibroblasts) (1208),[3] C- $C_6H_5$ str. Trp, Phe (1208),[5,23,41,42,76] A,T (ring breathing modes of the DNA/RNA) (1208),[1,18] A, T, ring breathing of DNA/RNA (hematopoietic cell) (1208),[1] p-Cymene ring def. (monocyclic monoterpenes) (1208),[6] C-$C_6H_5$ str. Trp, Phe (1209),[4,17-19] Phe (thymus chromatin) (1209),[7] C-$C_6H_5$ str. Phe, Trp (ricin and sulfur mustard toxicity in lung cell, epithelial cancer) (1209),[4,19] C-$C_6H_5$ str. in Tyr, Phe (R17-virus) (1210),[18,22,77] human RBC (1210),[18,29] dopamine transporter (1210),[37] T (breast normal and cancer cells and prostate cancer cells) (1210)[21] |
| **1279.6** | lipid (breast cancer) (1277),[64] thymus chromatin (1277),[7] collagen (carcinogenesis of oesophagus) (1278),[12,16,18] $\alpha$-helix (epithelial cancer) (1279),[4,18] $CH_2$ (lipids, human colon cancer) (1280),[53] $CH_2$ wag. from glycine backbone & Pro side chains (1280),[18,30] collagen (1280),[18,50] nucleic acids and phosphates (1280)[18,50] |
| **1344.7** | nuclear nucleic acid (Keratin-8/18 knock-down cells) (1342),[47] G (d18 stat mESC-CMs, d20 stat hESC-CMs, PC-4) (1342),[27] A (R17-RNA virus) (1342),[22] CH def., G (DNA/RNA) (1342),[18,19] HEK293 (dopamine transporter in cell) (1342),[37] CH def, A, G (ricin and sulfur mustard toxicity in lung cell) (1342),[19] A, Trp (R17-virus) (1343),[22] $CH_3$, $CH_2$ wag. (collagen, human breast tissue) (1343),[18,28] Glucose (1343),[18,48] CH def. (1343, 1344),[18,52] nuclear region (keratin-8/18 knock-down cells) (1345),[55] $CH_3CH_2$ wag. (collagen, epithelial cancer) (1345)[4] |
| **1427.4** | A, G (IRPT/J800) (1427),[10] $CH_2$ bend. and lipids (collagen) (1428-1455),[57] cellulose $CH_2$ def. (polysaccharides) (1430)[6] |
| **1454.7** | polysaccharides (MCF10A/MDA-MB-435) (1452),[51] $CH_2$,$CH_3$ sciss., def (lipid, breast cancer) (1452),[64] $CH_2$ (thymus chromatin) (1452, 1453),[7] benign and malignant tissues (breast cancer) (1452),[61] umbrella mode of methoxyl (1453),[6,18,78] C-H bend. (1453),[18,71] tumors (1453),[18,62] $CH_2$ str., $CH_3$ assym. def.[18,43] asym. $CH_3$ bend.,$CH_2$ sciss. (elastin, collagen, and phospholipids) (1454),[18] collagen and phospholipids (1454),[18,62] $CH_2$ def. ($\beta$-carotene, tetraterpenes) (1454, 1455),[6,18,52] $CH_3$ asym. bend.,$CH_2$ sciss. (cancerous tissue of the nasopharynx) (1454),[77] deoxyribose (1455, 1457),[18,33] nucleic acid, Keratin-8/18 knock-down cells (1457)[55] |
| **1488.8** | G, A (CHL-cell metaphase chromosomes) (1487),[35] G (N7) (1487),[18,33] HEK293 (dopamine transporter) (1487),[37] G (N7) (1488),[18,33] collagen (1488),[18,79] A, G, ring modes (inner histones bound to DNA in chromatin) (1489),[14] DNA (1490),[18,59] normal and tumor tissues (1490),[18,76] G, A (thymus chromatin) (1490)[7] |
| **1518.5** | C-C str. ($\beta$-carotene) (1517),[18,71] C=C str. (capsanthin, capsicum annuum L., tetraterpenes pepper) (1517),[6] A, C, G (IRPT/J804) (1518),[10] C=C str. (porphyrin) (1518),[18,41] C-C & C=C str. (Carotenoid) (1518),[18,41] cis-bixin C=C str. (tetraterpenes) (1518),[6] carotene (1520),[18,59] -C=C- carotenoid (epithelial cancer) (1520-1538)[4,18] |
| **1577.0** | G, A (R17-virus) (1575),[22] G, A, ring breathing modes (hematopoietic cell) (1575),[1,18] nucleic acid mode in tissues (1576),[42,69,70] G ($N_3$) (1576),[18,33] CC str. (4-mercaptobenzoic acid in cells) (1576),[73] G, A (CHL-cell metaphase chromosomes) (1577),[35] DNA: A, G, C=C, N-H def., C-N str. (cancerous murine fibroblasts) (1577),[3] G ($N_3$) (1577, 1578),[18,33] NADH (1577),[18,59] IgG (1577),[18,52] A, (1578),[27] PC-4 (1578),[27] C, G, A (ricin and sulfur mustard toxicity in lung cell, IRPT/J806) (1578),[10,18,19] pyrimidine ring (nucleic acids, heme protein, epithelial cancer) (1579),[4,17,18] Phe and Trp (DA-HEK293, dopamine transporter) (1579-1582),[37] Phe and Trp (dopamine transporter) (1579-1582),[37] DNA bands (cancer cell - U87-MG glioblastoma cells) (1579) [54] |
| **1634.1** | C=C str.($\beta$-caryophyllene, sesquiterpenes) (1632),[6] C=C str. (myrcene, acyclic monoterpenes) (1634),[6] DC473 Ester, colorectal cancer cell (1634),[44] C=C str. ($\beta$-caryophyllene) (1635)[6] |
| **1656.8** | C=C str. (collagen) (1654),[28,30,42,80] C=O str., α-helix conformation, C=C str. (lipid, epithelial cancer) (1654, 1655, 1655-1680),[4,12,17,19,23,41,42,76,81,82] collagen (1654, 1655),[5,28,42,62,83] hDAT-HEK293 (dopamine transporter) (1654),[37] human breast tissue (1654),[28]MCF-7 (1655),[46] collagen (1655), C=C (lipids) (1655),[18,59] T, G, C (ring breathing modes) (1655–1680),[1,18] T, G, C ring breathing modes (hematopoietic cell) (1655),[1] C=C lipid (oral squamous cell carcinoma) (1655),[24] C=C (unsaturated lipids) (1655),[47] N-C=O (lipid) (1655),[47] $\alpha$-Helix, C=O str., N-H wag. (1655),[6] C=C str., (sabinene, bicyclic monoterpenes) (1655),[6] C=C (unsaturated lipids) (1655),[47] N-C=O (lipids) (1655),[47] human breast tissue (1655),[28] C=C str. (lipids, ricin and sulfur mustard toxicity in lung cell) (1655-1680),[19] C=O str. α-helix conformation, C=C str. (lipid, epithelial cancer) (1655),[4] C=O str. of carbonyl groups (melanocytes) (1655),[32] inner histones bound to DNA in chromatin (1656),[14] cancerous murine fibroblasts (1656),[3] C=C (lipids) (1656),[18,52] C=C str. (cis-phospholipids) (1656),[18,52] thymus chromatin (1656),[7] inner histones bound to DNA in chromatin (1657),[14] fatty collagen (1657) [28,30,42,61] triglycerides, fatty acids, breast Cancer (1657),[42,61,71] human breast tissue (1657),[28] PC-1, PC-5, d20 dyn hESC-CMs (1658),[27] maturation of CMs (1658),[27] α-helix (1658),[18,52] C=C str. ($\alpha$-pinene, bicyclic monoterpenes) (1658),[6] thymus chromatin (1658)[7] |
| **2133.0** | azide moieties (-$N_3$) (breast cancer) (2120),[45] C-D str. (D7-Glc & CD-AA) (213),[46] C-D bond (lipid) (2133)[47] |
| **2152.0** | CD mode vimentin (2150)[84] |

(v) NIH Component 1

| ($\tilde{\nu}_{obs}$) | Assignment (reported $\tilde{\nu}_{obs}$) |
|---|---|
| **248.3** | unassigned |
| **651.0** | 1,8-cineol/sabinene ring def. (bicyclic monoterpenes, eucalyptus) (652) [6] |
| **995.8** | amphetamine content (994),[23] C-O ribose, C-C (996),[18,30] =C-O wag., piperine of ground black pepper (996),[6] $CH_2$ rock. (indocyanine green) (996),[10] IRPT/J779 (998)[10] |
| **1168.0** | Pro (human breast tissue) (1166),[28] N= Quinoid ring, ==N str. and C-H in plane bend. (1167),[18,29] lipids (1168),[18,30]C=C str. COH def. (lipid) (1168),[18,23] C-C str., carotenoid (1168),[18,31] Tyr. (collagen) (1169),[5,18] Phe (1170)[51], C-H in-plane bend. of Tyr (epithelial cancer) (1170),[4,17,18] nuclei include the O-P-O str. (melanocytes) (1170)[32] |
| **1233.8** | O-P-O asym. str. (melanocytes) (1232-1236),[32] nucleosome treated by $\alpha$-chymotrypsin (1233),[15] inner histones bound to DNA in chromatin (1234),[14] A concerted ring mode (1234),[9,18] DA-hDAT-HEK293 (dopamine transporter) (1234),[37] keratin (MCF10A/MDA-MB-435) (1235),[51] disordered structure (non-hydrogen bonded) (1235)[6] |
| **1286.8** | lipids, $CH_2$, $CH_3$ def. (IRPT/J794) (1286),[10] cytosine (1287),[18,33] DA (dopamine transporter) (1287),[37] phosphodiester groups in nucleic acids (1286) [18,30] |
| **1330.6** | keratin-8/18 knock-down cells (1329),[85] phospholipids (1330),[42,59,62] collagen (1330),[18,50] nucleic acids and phosphates (1330),[18] $CH_2$ twist. $CH_2$ bend. (lipid, oral squamous cell carcinoma) (1330),[24] DA-hDAT-HEK293 (dopamine transporter) (1331),[37] -C str. of Phenyl and C-C str. (1332) [6,18] |
| **1390.3** | dopamine transporter (1390),[37] collagen (1390),[57] CH rock. (1391),[6,18] C-N str., benzoid ring, in quinoid ring (1392)[18,29] |
| **1430.3** | $CH_2$ bend. (lipids, collagen) (1428-1455),[57] cellulose $CH_2$ def. (polysaccharides) (1430)[6] |
| **1511.6** | Adenine (CHL-cell metaphase chromosomes) (1510),[35] cytosine (1510),[18,33] A (ring breathing modes, hematopoietic cell) (1510),[1,18] C=C str. (lycopene, tetraterpenes) (1510),[6] C=C bonds (1510),[6] A, thymus chromatin (1512),[7] C (1513)[18,33] |
| **1587.1** | Asp, Glu C=O str (cancerous murine fibroblasts) (1585),[3] C=C olefinic str. (1585),[23,42,43] cytochrome C peak (colorectal cancer cell) (1585),[44] Phe, hydroxyproline (1586),[18 5] DA-hDAT-HEK293 (dopamine transporter) (1586),[37] Trp (MCF10A/MDA-MB-435) (1587),[51] Phe, hydroxyproline (1588),[5,18] C=C str. (pMBA) (1588)[86] |
| **1617.7** | C=C str. Tyr., Trp, C=C (hematopoietic cell, cancerous murine fibroblasts, epithelial cancer) (1615, 1616),[1,3,4,17,42] C-C str. (human RBC) (1617),[18,29] C=C str. Phe, Tyr (1617),[18,19] hDAT-HEK293 (dopamine transporter) (1617),[37] dopamine transporter (1617),[37] Tyr, Phe (thymus chromatin) (1617, 1618),[7] C=C Tyr, Trp (ricin and sulfur mustard toxicity in lung cell) (1617),[19] C=C str. Trp (1618),[18,41] C=C str. (porphyrin) (1618),[18,41] NADH (1618),[18,59] heme aggregation, Fe atom in a low - spin state (S=0) (human erythrocytes) (1618)[38] |
| **1998.8** | unassigned |
| **2163.7** | CD mode (vimentin) (2150)[84] |
| **2322.3** | region of the OH-NH-CH str. (2330-3800)[18,87] |
| **2502.9** | unassigned |
| **2839.2** | $CH_3$ sym. str. of lipids (2840-2875)[18,63] |

(vi) NIH Component 2

| ($\tilde{\nu}_{obs}$) | Assignment (reported $\tilde{\nu}_{obs}$) |
|---|---|
| **281.5** | unassigned |
| **394.8** | phosphatidylinositol (415)[18,48] |
| **455.6** | ring torsion of phenyl-2 (454),[6,18] S-S bridge (human lymphocyte) (458-567)[34] |
| **513.7** | interchain S-S bridge (human lymphocyte) (510),[34] S–S str., cysteine, g–g–g (512)[6] |
| **544.1** | S-S str. g-g-t, cysteine (540),[12,18] glucose-saccharide band (540),[18,48] cysteine, S–S str., t–g–t (540),[6] anthocyanidin mono-glycosides (540),[6] cholesterol (548)[18,48] |
| **582.7** | G, C (R17-RNA virus) (580),[22] OH out of plane bend. (583)[6,18] |
| **607.6** | glycerol (607),[18,48] cholesterol (608)[18,48] |
| **650.5** | 1,8-cineol ring def. (bicyclic monoterpenes, eucalyptus) (652),[6] sabinene ring def. (bicyclic monoterpenes) (652)[6] |
| **674.8** | T, G (d(CGCGAATTCGCG)) (672),[2] oligomer, T, G, furanose rings (672),[2] C-S str. (colorectal cancer cell) (674)[44] |
| **701.6** | phosphatidylcholine, lipids (breast normal and cancer cells and prostate cancer cells) (699),[21] C–S str. Met (700-745),[2,12,42] cholesterol, cholesterol ester (702)[18,48] |
| **723.7** | A (R17-virus) (722, 725),[22] DNA (722),[16,18] DNA, carcinogenesis of oesophagus (722),[12] C-S, C-C (cancerous murine fibroblasts) (724),[3] A ring breathing mode of DNA/RNA bases (hematopoietic cell) (725)[1,18] |
| **751.3** | sym. breathing of Trp (749),[5,17,18,41] dopamine transporter (749),[37] T (CHL-cell metaphase chromosomes) (750),[35] $CH_{2,6}$ out-of-plane bend.(human RBC) (750),[6,18] lactic acid (carcinogenesis of oesophagus) (750, 752),[12,16,42] nucleic acids, O-P-O sym. str. (human lymphocyte) (750),[34] T (thymus chromatin) (751, 752),[7] porphyrin breathing mode,(RBC & heme groups of the hemoglobins) (752),[18]DNA (752),[18,75] sym. breathing of Trp (752, 753),[5,17,18,41] $CH_2$ rock., sym. breathing, Trp (cytochrome c, mitochondria) glioblastoma multiforme (752)[67] |
| **784.5** | dodecamer in solution (782-783),[2] DNA (782),[18,19,75] U, T, C (ring breathing modes) (782),[18,19] carcinogenesis of oesophagus (782),[12] C (d(CGCGAATTCGCG)) (783),[2] sym. phosphodiester str. and ring breathing modes of pyrimidine (783-790),[47] 5',3'-deoxyribosephosphate backbone (784),[8] phosphodiester; cytosine (784, 785),[18,33] phosphodiester bonds of DNA, nucleic acid specific band(d18 stat mESC-CMs, d20 stat hESC-CMs, PC-4) (785),[27] U, T, C, ring breathing modes backbone O-P-O (785),[1,18] T, C backbone O-P-O, (nucleus-cytoplasm, |

| | |
|---|---|
| | CHO cells) (785),[68] U, T, C ring breathing modes, O-P-O (hematopoietic cell) (785),[1] (cancer cell,U87-MG glioblastoma cells) (785),[54] C, T, P (nucleosome treated by α-chymotrypsin) (785)[54] (787),[6] phosphate furanose backbone (786),[2] crystalline dodecamer (786),[2] d(CGCGAATTCGCG) (786),[2] DNA: O-P-O, C, U, T (epithelial cancer) (786),[4,18] pyrimidine ring breathing mode (786),[9,18]C, U (R17-RNA virus) (787),[22] phosphatidylserine (787),[18,48] nucleic acids, C, U, T (microbial cells) (787),[29] α-pinene wag., C-H str.(bicyclic monoterpenes) (787)[6] |
| **842.5** | α-anomers (840),[18,48] glucose-saccharide band (840),[18,48] polysaccharide structure (840-860),[11,18] glucose (epithelial cancer) (842),[4,18] 1,8-cineol C-H wag. (bicyclic monoterpenes) (843)[6] |
| **906.1** | IRPT/J777 (906)[10] |
| **935.3** | DNA (cancerous murine fibroblasts) (933),[3] proline, hydroxyproline, C-C str. skeletal of collagen backbone (933),[5,18] CC skeletal str., α-helix, C-C str. (cancerous murine fibroblasts) (934),[3] C-C backbone (collagen, human breast tissue) (934),[28] C-C str. mode of Pro (935),[18,41,88] Val (α-helix, glycogen) (935),[4,18] P($CH_3$) terminal, Pro, Val CC str. α-helix (keratin, epithelial cancer) (935),[18,23] keratin (MCF10A/MDA-MB-435) (936),[51] DA-hDAT-HEK293 (dopamine transporter) (936),[37] R (thymus chromatin) (936),[7] hydroxy Pro (collagen) (936),[57] Pro (collagen), hydroxyproline, C-C str. (937),[5,18] C-C backbone (collagen) (937),[18,28] glycogen (937),[16,18] C-C str., α-helix, C-O-C glycosides (937),[19,42] Pro, hydroxy Pro, C-C str. (collagen) (937),[5,18] C-C, backbone (human breast tissue) (937),[28] C-C str. α-helix, C-O-C glycos. (carbohydrates) (ricin and sulfur mustard toxicity in lung cell) (937),[19] glycogen peaks (carcinogenesis of oesophagus) (937)[12] |
| **969.6** | hDAT-HEK293 (dopamine transporter in cell) (967),[37] lipids (968),[18,30] phosphate monoester groups of phosphorylated (970),[18,30] C-O str. (melanocytes) (970),[32] C-C wag. (971)[18,52] |
| **1035.9** | collagen(1033),[18,30] Phe (1033),[1,18] CO str., CC str., CCO str. (polysaccharides, pectin, cellulose) (1033, 1035),[6,12,18] C-H in-Phe (1033),[18,19] C-C str. (lipid) (1033),[86] CO str., CC str., CCO str. (pectin, polysaccharides) (1033),[6] Phe (thymus chromatin) (1033),[7] C-H in-plane Phe (ricin and sulfur mustard toxicity in lung cell) (1033),[19] Phe (collagen) (1034, 1045),[5,18,50] ribose, Phe (R17-virus) (1035),[22] hDAT-HEK293 (dopamine transporter) (1035) [37] |
| **1077.4** | alkyl C-C g str. (lipid) (1075, 1076),[20] C-C chain str. (lipid, phospholipids, membrane CHO cells) (1075),[42,59,68] sym. str. of $PO_4^{3-}$ (1076),[5,18] DA-hDAT, α($H_{19}$-$N_9$-$C_8$, $C_7$-$C_8$-$H_{18}$), γ($N_9$-$H_{19}$, $C_8$-$H_{19}$, $N_9$-$H_{20}$, $C_8$-$H_{18}$), DA-hDAT-HEK293, (dopamine transporter) (1076),[37] C-C or C-O str. (phospholipids, lipid, epithelial cancer) (1078), [4,18,41] sym. phosphate str. (1078),[18,41] phospholipids (1078),[18,41] C-C or C-O str. (phospholipids) (1078),[18,41] CC skeletal (1078),[18,23] CC str., $PO_5$ (nucleic acid) (1078),[18,31] breathing vibration of the aromatic ring (pMBA) (1078),[86] normal breast tissue (1078),[61] Pro (human breast tissue) (1079)[28] |
| **1106.5** | C-N str (thymus chromatin)(1103),[7] Phe (1104),[52] carbohydrates (1105),[30] CN str. (IRPT/J782) (1106),[10] CO, CC str. ring (pectin, polysaccharides) (1107),[6] $NH_3^+$ asym. rock. of His (hDAT-HEK293, dopamine transporter) (1108-1113)[37] |
| **1126.1** | C-C str. skeletal of acyl backbone (lipid) (1124),[5,18] (polysaccharides, MCF10A/MDA-MB-435) (1125),[51] R17-virus (1125),[22] CO, CC str., ring (disaccharides, sucrose, cellulose, polysaccharides, (1125, 1126),[6,12,18] Pro. (human breast tissue) (1125),[28] inner histones bound to DNA in chromatin (1126),[14] paraffin (1126),[18,30] C-C str. skeletal of acyl backbone in lipid (trans-conformation) (1126, 1128),[5,18] C-N str.(1126),[1] C-N str. (hematopoietic cell) (1126),[1] C-N str. (1127),[18,52] Pro. (human breast tissue) (1127),[28] CN, CC, CO str. skeletal (cancerous murine fibroblasts, (ricin and sulfur mustard toxicity in lung cell) (1128)[3,19,42] |
| **1156.9** | CC, CN str., $CH_3$ rock. (IRPT/J785) (1154),[10] C-C str. cis-bixin (tetraterpenes) (1154),[6] C-C, C-N str., (carotenoids, epithelial cancer) (1155, 1156, 1157),[4,17,18,69] glycogen (1155),[18,30] C-C str. (carotenoid, cellular pigment) (1155),[18,29] C-C str. (microbial cells) (1155),[29] C-C str. trans-bixin (tetraterpenes) (1155),[6] C-C, C-N str. (1156),[1,18] carotenoids (1156, 1157),[18,69]C-C, C-N str. (hematopoietic cell) (1156),[1] C-C str. (lycopene, tetraterpenes) (1156),[6] CN, CC str. (cancerous murine fibroblasts) (1157),[3] in-plane vibrations of the conjugated (1157),[18,89] C=C str. (ß-carotene )(1157),[18,71] C-C str. (lutein, tetraterpenes, yellow root) (1157),[1] T, G (nucleosome treated by α-chymotrypsin) (1157),[15] C-C, C-N str. (1158),[18,19] C-C str. (capsanthin) (1158),[1] acetoacetate (breast normal and cancer cells and prostate cancer cells) (1158),[21] C-C, C-N str. (ricin and sulfur mustard toxicity in lung cell) (1158)[19] |
| **1178.1** | C, G (1176),[18,33] C-H bend. (1176),[18,19] Tyr (ricin and sulfur mustard toxicity in lung cell) (1176),[19] C, G (1177),[18,33] Tyr, Phe (thymus chromatin) (1177),[7] C, G, A (R17-RNA virus, epithelial cancer) (1180),[4,22,33,42] C, A (1180-1184),[4,18] C-C-O str. (1180-1260)[6] |
| **1227.1** | melanocytes (1225-1242),[32] O-P-O asym. str. (melanocytes) (1225-1260, 1230, 1230-1240),[32] geranyl acetate C–O–C str. (1227),[1] asym. phosphate str. (1230),[18,30] N-H , C-N str. (1230-1300),[18,30] ß-sheet (breast normal and cancer cells and prostate cancer cells) (1230)[21] |
| **1254.2** | inner histones bound to DNA in chromatin (1252),[14] G, C ($NH_2$) (1252),[18,33] C-O aromatic str. (1252),[6,18] thymus chromatin (1252-1253),[7] T, C, A, ring str., $CH_2$, $CH_3$ def. (IRPT/J791, lipids) (1254),[10] adenine (CHL-cell metaphase chromosomes) (1254),[89] C-N in-plane str. (1254),[18,29] C,G containing oligomers and polymers (1255),[8] lipids (1255),[18,30] C, T (nucleosome treated by α-chymotrypsin) (1255),[15] reactive (benign breast cancer) (1256),[64] -$CH_2$ rock. (1256)[6] |
| **1309.5** | C-N asym. str. of aromatic amino acids (1307),[85] $CH_3$, $CH_2$ twist., bend., wag (lipid/collagen) (1307),[5,18] C-N asym. str. (1308),[29] ring str. of Phe (dopamine transporter) (1308-1310),[37] phospholipid (cancer cell - U87-MG glioblastoma) (1308),[54] $CH_3$/$CH_2$ twist. or bend. (aromatic amines of lipid/collagen) (1309),[5,18] C-N str., N-H bend. (DA-hDAT-HEK293, dopamine transporter in cell) (1311 -1312)[37] |
| **1322.9** | CH def. (cytoplasmic region of keratin-8/18 knock-down cells) (1320),[55] G (R17-RNA virus) (1320),[22] CH def., G (DNA/RNA, ricin and sulfur mustard toxicity in lung cell) (1320),[18,19] α-helix (1321),[18] $CH_3CH_2$ twist. (collagen, nucleic acids) (1322),[18,41] G (B,Z marker) (1322),[18,33] $CH_3CH_2$ wag. (collagen & purine bases of DNA) (1324)[18,77] |
| **1390.4** | dopamine transporter (1390),[37] collagen (1390),[57] CH rock. (1391),[6,18] C-N str. in quinoid ring-benzoid ring (1391)[18,29] |
| **1430.1** | $CH_2$ bend. protein and lipids (collagen) (1428-1455),[57] cellulose $CH_2$ def., polysaccharides (1430) [6] |

| | |
|---|---|
| **1450.4** | Trp, $CH_2$ def. (keratin, MCF10A/MDA-MB-435) (1448),[51] $CH_2$, $CH_3$ def. (lipids, proteins, IRPT/J801) (1448),[10] $CH_2CH_3$ def., $CH_2$ def., -$CH_2$ bend. (collagen, tumor) (1448),[5,6,28,42,59,90] CH def. (CHL-cell metaphase chromosomes) (1449),[89] C-H str. (lipids) (1449),[18,19] $CH_2$ bend., nucleic acid (cytoplasmic region of keratin-8/18 knock-down cells) (1450),[85] $CH_2$ def., $CH_2$ str. (vimentin) (1450),[84]C-H def. (R17-virus) (1450),[22] $CH_2$ def., (cancerous murine fibroblasts) (1450),[3] $CH_2$ bend. (malignant tissues) (1450),[42,59,80,82,91] $CH_3$ bend. Methylene def. $CH_2$ def. (collagen, IDC breast tissue) (1450),[18,30] [18,28] C-H def. (membrane-cytoplasm-nucleus, CHO cells, lipids) (1450),[18,29,68] $CH_2$ bend. (1450),[18,52] CH def. (DNA assignment), CH def. (lipid assignment), expected location: membrane-cytoplasm-nucleus (CHO cells) (1450),[68] $CH_2$ def. (nucleic acid (tumor cells) lipids, human lymphocyte) (1450),[34] C-H def. (microbial cells) (1450),[29] C-$H_2$ (lipid, nucleic acid, tumor cells) (1450),[26] $CH_2$ def., thymus chromatin (1450, 1451, 1452),[7]$CH_3$, $CH_2$ def. (human breast tissue) (1450, 1451), [28,92] infiltrating ductal carcinoma, benign and malignant tissues, breast cancer (1450, 1452),[61] C–$H_2$ twist (lipid, tumor cell) (1450),[26] C-H def. (aliphatic amino acids, inner histones bound to DNA in chromatin) (1451),[14] $CH_2CH_3$ def. (collagen assignment) (1451),[18,28,52] dopamine transporter (1451),[37] polysaccharides (MCF10A/MDA-MB-435) (1452),[51] $CH_2$,$CH_3$ sciss., $CH_2$, $CH_3$ def. (cancer cells, collagen) (1452)[64] |
| **1550.1** | IRPT/J807 (1548),[10] Trp (epithelial cancer) (1548),[4,17,18] DNA repair, melanocytes (1549),[32] Trp (R17-virus) (1550),[22] lipid, oral squamous cell carcinoma (1550),[24] Trp (1552),[18,41] C=C str., Trp (1548)[18,41] |
| **1583.1** | heme aggregation, Fe atom in the high-spin (S=0, 2) (human erythrocytes) (1581, 1582),[38] Phe, Tyr (IRPT/J809) (1582),[10] def C==C, Phe (1582),[18,41] C=C bend. of Phe (1583),[18,77] Trp and C=C bend. (cancerous tissue of the nasopharynx) (1583),[39] malignant tissue (bladder cancer in urine) (1584),[60] Asp, Glu C=O str. (cancerous murine fibroblasts) (1585),[3] C=C olefinic str. (1585),[23,42,43] cytochrome-c peak (colorectal cancer cell) (1585) [44] |
| **1619.1** | CC str. (human RBC) (1617),[18,29] C=C Phe, Tyr (ricin and sulfur mustard toxicity in lung cell) (1617),[18,19] hDAT-HEK293 (dopamine transporter) (1617),[37] Tyr, Phe (thymus chromatin) (1617, 1618, 1621),[7] C=C str., Trp (porphyrin) (1618),[18,41] Trp (1618),[18,41] NADH (1618),[18,59] heme aggregation, heme Fe atom in a low - spin state (S=0) (human erythrocytes) (1618),[38] U, Trp, Tyr, Phe (R17-RNA virus) (1620),[22] C=C str. (porphyrin) (1620),[18] in-plane double end vibrations of bases (1620-1750),[18,30] cytoplasm-nucleus, CHO cells (1620),[86] C=C str. (lipid) (1620)[86] |
| **1721.5** | C=O str. (cortisone) (1716-1741),[18,48] C=O str. (citronella, acyclic monoterpenes, eucalyptus) (1725)[6] |
| **2130.2** | azide moieties (-$N_3$) (breast cancer) (2120),[45] C-D vibration (D7-Glc & CD-AA) (2133),[46] C-D bond (lipid) (2133)[47] |
| **2232.5** | -CN str. (breast cancer) (2230)[45] |

**Table S2.** The list of Raman spectral lines ($\tilde{\nu}_{obs}$) in cm$^{-1}$ selected from **Fig. S2**. Here, str. = stretching, sym. = symmetric, and asym . = asymmetric, wag. = wagging, and twist. = twisting. The abbreviations of amino acids are: G: Guanine, C: Cytosine, T:Thymine.

| No. | Raman Shift | Spectral Assignment (summarized from **Table 1**) |
|---|---|---|
| [1] | 2131–2153 cm$^{-1}$ | azide moiety, vimentin |
| [2] | 1872 cm$^{-1}$ | unassigned |
| [3] | 1722 cm$^{-1}$ | C=O str. |
| [4] | 1105–1250 cm$^{-1}$ | CN str., $NH_3$ asym. str. |
| [5] | 890–1000 cm$^{-1}$ | RNA $PO_4^-$ sym. str., C–O–C skeletal modes |
| [6] | 810–845 cm$^{-1}$ | RNA, O–P–O str., C–O–C skeletal modes |
| [7] | 651–675 cm$^{-1}$ | S=O, C–S str., CO wag., CC twist., T, G |
| [8] | 596 cm$^{-1}$ | T, C, G ring modes |
| [9] | 549–580 cm$^{-1}$ | SS str. |
| [10] | 257 cm$^{-1}$ | unassigned |

**Table S3.** The list of major Raman peaks ($\Delta\tilde{\nu}$) based on difference spectrum shown in **Eq. (S1)** focusing on spectral lines mainly observed in Component 2, which are considered to be important to conform outside or connect the cell frame work are listed for (i) A431, (ii) A431D, and (iii) NIH. The assignments are given with observed wavenumbers in parenthesis. Here, def. = deformation, str. = stretching, sym. = symmetric, and asym. = asymmetric, wag. = wagging, sciss. = scissoring, and twist. = twisting. Gauche form is given by g, and Trans form is given by t. The abbreviations of amino acids are: Tyr: Tyrosine, Leu: Leucine, Gly: Glycine, Ala: Alanine, Met: Methionine, Phe: Phenylalanine, Gln: Glutamine, Lys: Lysine, Arg: Arginine, Asp: Asparagine, Val: Valine, Ile: Isoleucine, Glu: Glutamic acid, His: Histidine, Cys: Cysteine, and Trp: Tryptophan. A: Adenine, G: Guanine, C: Cytosine, T:Thymine.

(i) A431 Cell

| $\Delta\tilde{\nu}$ ($cm^{-1}$) | Assignment |
|---|---|
| **289.0** | unassigned |
| **308.5** | unassigned |
| **458.8** | [1] S-S bridge[34] |
| **553.0** | S-S bridge,[34] S-S str.[40] |
| **636.7** | disulfide, S-S, C-S bond,[22] C-S str., Tyr. C-C twist.[1] |
| **642.9** | [2] C-S str., C-C twist. Tyr., Phe.[1,3-7,19] A, G, deoxyribose phosphate backbone[2,19] |
| **664.4** | Cys. C-S str. [5,17,34] Tyr.-G backbone in RNA,[1] T, G[19] |
| **676.6** | [3] T, G ring breathing modes, oligomer [1,2,4] C-S str.,[44] proteins[21] |
| **761.8** | Trp., ring def., ring breathing, A, T[4,6,12,19,22] ethanolamine group, sym. str. $N^{+}(CH_3)_3$[48,86] $CH_2$ rock.[34] |
| **786.0** | C, phosphodiester,[2,33] sym. str. phosphodiester and ring breathing modes of pyrimidine bases,[47] U, T, C, P (ring breathing modes in the RNA bases) backbone O-P-O, pyrimidine ring breathing mode nucleic acids,[1,4,7,9,15,22,29,35] nucleus-cytoplasm,[68] C–H wag.[6] |
| **831.0** | [4] phosphodiester,[33] O-P-O ($PO_2^-$) asym. str., RNA, Tyr. out-of-plane ring breathing, Pro., hydroxyproline,[4-7,12,19,22] $CH_2$ wag.,[6] C-H out of plane bend. in benzenoid ring,[13] P, ($PO_3^{2-}$), $PO_4^{2-}$ (831)[15] |
| **908.4** | [5] amino acids[10] |
| **1034.3** | C-H def., Phe., C-H in-plane bend. and str. C-N str., C-C skeletal str., $CH_2CH_2$ bend., C-C ring in aromatic structure of xylene, Pro[1,3-5,7,17,19,28,34,41,43] CO str., CC str., CCO str.[6,12,86] |
| **1117.9** | $CH_{2,6}$ in-plane bend.,[6] Pro.,[28] proteins C-C str., def. C-O-H., C-O str., CN str.[4,10,20,26,30,47] |
| **1189.2** | asym. str. phosphate,[50] COC str.,[6] CN str. Tyr., Phe.[10] |
| **1225.9** | [7] $PO_2^-$ str.,[41] A concerted ring mode,[9] proteins,[12,16] Amide III, coupling of C-N str. and N-H bonding, ß-sheet structure,[30,52] asym. str. O-P-O ($PO_2^-$),[30,93] C–O–C str.[6] |
| **1279.2** | Amide III, α -helix, $CH_2$ wag., Pro. side chains,[4,7,30,64] proteins,[12,16,53] nucleic acids and phosphates[50] |
| **1307.1** | [8] $CH_2$, $CH_3$ twist. bend, wag. str. and/or bend., Amide III, N-H bend., α-helix, C-N asym. str., phospholipids, protein, (aromatic amino acids, aromatic amines)[5,7,28,29,54,55,67] $CH_2$ def., A, C,[4] $COO^-$ sym. str.,[37], Phe. ring str., phospholipid[37] |
| **1346.0** | [9] A, Trp.,[22] $CH_2$, $CH_3$ wag.,[19,28] CH def.,[52] $CH_2$, $CH_3$ wag.[4] |
| **1365.3** | G, Trp.,[5,22,33] sym. def. $CH_3$ C=O,[6] pyrrole in-phase breathing modes[38] |
| **1368.0** | [10] G, Trp.,[4,5,22,33] $CH_3$ sym. str., $CH_3$ sym. def., C=O sym. def., phospholipids,[6,52] pyrrole in-phase breathing modes,[38] $CH_2$ def., CC str.,[6,53] |
| **1488.3** | [12] $NH_3^+$,[43] G, A,[1,4,7,33,35] C-N str. coupled with the in-plane C-H bend. in amino radical cations[43] |
| **1504.5** | In-phase C=C str. benzenoid ring,[6,29] A, C, G,[10,33] =N-H bend.[29] |
| **1544.8** | [13] C-C,[29] C-H def.,[9] Trp.[4,10,17,30,93] |
| **1577.0** | [14] A, G, C=C, N-H def., C-N str. (Amide II), G ($N_3$), A, Phe., Trp., nucleic acid mode,[3,4,7,10,19,22,27,33,35,37,69,70] CC str.,[29,73] pyrimidine ring (nucleic acids),[4,17] aromatic and aliphatic -C=C- and >N-C=O str.,[6] breathing mode[73] |
| **1603.6** | protein,[22] Amide I, H- bonding of peptide, nucleic acids, C=O, -C=C bend. def., benzene ring C-C str., Amide I α-helix, CO str. C=C in-plane bend., Phe. Tyr., unsaturated fatty acids, aryl ring, ring C-C str. of Phe., Tyr., C=C[1,3-6,10,15,17,19,20,36,41,47,52,67,69,93] Cyt. $NH_2$,[33] pectin $COO^-$ str.[6] |
| **2187.5** | azide moieties, $-N_3$ (breast cancer)[45] |
| **2371.4** | [15] unassigned |
| **2508.7** | unassigned |

(ii) A431D Cell

| $\Delta\tilde{\nu}$ ($cm^{-1}$) | Assignment |
|---|---|
| **200.9** | unassigned |
| **259.8** | unassigned |
| **321.4** | unassigned |
| **411.2** | phosphatidylinositol, cholesterol[48] |
| **664.4** | [2] C, C-S str.,[5,17,34] Tyr.-G backbone,[1] ring def.,[6] T, G[2,19] |
| **810.0** | sym. str. di-oxy of the phosphate backbone from nucleic acids and phospholipids, phospho-diester, O-P-O str. RNA[1,19,33,53] C-C str.,[28] def. C-H[6] |
| **837.0** | [4] O-P-O asym. str., Tyr., RNA, protein, C-O and C-P-O str. in the nucleic acid backbone,[1,7,34,93] phosphate furanose backbone,[2] def. amine groups,[29] C-H wag.[6] |
| **902.4** | [5] C-O-C skeletal str.,[6,12] di-oxy sym. str. of the phosphate backbone from nucleic acids and phospholipids,[53] amino acids[10] |
| **923.1** | Pro. C-C str. ring,[4,17,28] $CH_2$ wag.[6] |

| | |
|---|---|
| **999.4** | C-O, C-C, C-C aromatic ring str., CC str.,[30,43,75] =C-O wag.,[6] $CH_2$ rock.,[25] Amide III,[10] sym. dioxy str. of the phosphate backbone from nucleic acids and phospholipids,[53] Phe. aromatic ring, sym. ring breathing mode[3-5,17,28,51,59] out-of-phase C-C-O str.,[6] in-plane rock. $CH_3$ groups attached to the polyene chain and coupled with C-C bonds,[6] A, U, C[22] |
| **1166.4** | [6] C–O–C str., ring,[6] Tyr.,[5] C–C str., Pro.,[28,31] N= Quinoid ring, ==N str. and C-H in plane bend.,[29] C=C str., COH def., Tyr.[23] |
| **1203.3** | nucleic acids and phosphates,[50] Aromatic C-O and C-N,[72] Amide III, α-helix, β-sheet, C-N, N-H str.[1,19,20,26,59] bend. of electronic structure of nucleotides,[1] nucleic acids, phosphates, nucleotides base str. C-N, Tyr., Phe.,[3,4,10,28,52] $C-C_6H_5$ str., C-C ring in aromatic structure of xylene,[43] Pro. side chains, $CH_2$ wag. from glycine backbone,[30,55] Trp.[51] |
| **1293.2** | C-H bend.,[94] C, methylene, proteins,[3,33] $(CH_2)_2$ twist.,[6] methylene twist. [31] keratin,[51] $CH_2$ def.,[43] C-H twist.[57] |
| **1436.6** | [11] $CH_2$, $CH_3$, sciss. and def.,[4,6,28,43,48,52,59,61,63] acyl chains,[27,48] fatty acids,[51] $CH_2$ bend.[80] |
| **1488.3** | [12] $NH_3^+$, C-N str. coupled with the in-plane C-H bend. in amino radical cations,[43] G, A ring breathing modes, RNA[1,4,33,35] |
| **1518.0** | C,[33] C-C str.,[71] C=C str., C-C str.,[6,41] A, C, G[4,10] |
| **1583.0** | [14] C-C str.,[29] -C=C- olefinic str., >N-C=O str. (aromatic and aliphatic),[6,23,43] G, A,[7] ring breathing,[73] Phe., Tyr., Trp. C=C bend.,[10,39] C==C def.,[41] Asp, Glu C=O[3] |
| **1590.3** | Asp, Glu. C=O str.,[3] C=C olefinic str., C=N str. in quinoid ring (keratin-8/18 knock-down cells)[23,29,43,47,55,86] Trp.,[51] Phe., Tyr., hydroxyproline[5,10] |
| **1653.9** | N-C=O, cis-C=C str., C=O str., N-H wag., α helix, T, G, C ring breathing modes of the RNA bases, Amide I, vimentin,[1,4-7,9,12,14,17,19,23,24,28,30,41,46,47,52,59,76,80-84,90,93,95] |
| **2155.9** | CD mode (vimentin)[84] |

(iii) NIH Cell

| $\Delta\tilde{\nu}$ (cm$^{-1}$) | Assignment |
|---|---|
| **134.9** | unassigned |
| **191.0** | unassigned |
| **227.2** | unassigned |
| **279.3** | unassigned |
| **295.5** | unassigned |
| **388.8** | C[22] |
| **455.6** | [1] ring torsion of phenyl,[6] S-S bridge[34] |
| **652.0** | [2] A, G, deoxyribose phosphate backbone,[2] Tyr. C-C twist.,[19] G, Tyr.,[8] C, C-S str.[5,17] |
| **676.6** | [3] T, G oligomer, G associated with furanose rings, G ring breathing,[2,4] sym. str. C-S,[44] proteins[21] |
| **752.8** | $CH_2$ rock., Trp. sym. breathing,[5,17,41,67] T,[7,35] $CH_{2,6}$ out-of-plane bend., ring. def.,[6] nucleic acids, O-P-O sym. str.,[34] porphyrin breathing mode,[75] C[22] |
| **840.0** | [4] amine groups def.,[29] C-O, C-P-O str. in the nucleic acid backbone[93] |
| **917.2** | [5] Pro., hydroxyproline, C-C str. ring[3-5,17,28] |
| **934.9** | carbohydrates peak for solutions and solids,[30] skeletal C-C str., α -helix, Pro., hydroxyproline, C-C backbone str., C-O-C, Val. Backbone, α-helix, $P(CH_3)$ terminal, keratin[3-5,7,19,23,28,41,51,52,57,88] |
| **964.3** | $CH'_{2,6}$ out-of-plane bend.,[6] C=O def.,[6] wag. trans (RH)-C=C-(RH),[6] COH vibration[6] |
| **1086.3** | sym. str. O-P-O$^-$, $P(PO_3^{2-})$, $CO_3^-$, $PO_4^-$, C-C str. skeletal acyl group backbone, C-N str.[3-5,15,22,52,93,96] |
| **1126.5** | [7] Pro.,[28] C-C str. skeletal of acyl backbone trans-conformation, C-N str., C-O str., C-H str., ring, Acyl chains,[1,3-6,12,17,19,41,43,48,52,68] phospholipid trans versus gauche isomerism[61,97] |
| **1157.9** | C-C str., C-N str., in-plane vibrations of the conjugated, C=C str.,[1,3,4,6,17,19,20,29,71,89] T, G,[15] acetoacetate[21] |
| **1163.6** | [6] C-H str., quinoid ring def., N= Quinoid ring, ==N str., C-H in plane bend.,[6,20,29] C-O-C str. ring,[6] Tyr.,[5] Pro.,[28] C=C str., COH def.,[23] C-C str.[31] |
| **1225.9** | asym. str. O-P-O$^-$,[30,41,93] A concerted ring mode,[9] Amide III, ß-sheet, coupling of C-N str. and N-H str. mixed with side chains,[16,21,52] C-O-C str.[6,30] |
| **1254.0** | asym. str. $PO_2^-$,[93] G, C, $NH_2$,[33] C-O aromatic str.,[6] Amide III, A, T ring breathing C, G oligomers and polymers, ring str., proteins, $CH_2$, $CH_3$ def.,[1,2,7,10,15,34,35,64] C-N in plane str.,[29] $CH_2$ rock.,[6] keratin-8/18 knock-down cells[55] |
| **1312.7** | [8] $CH_3$, $CH_2$ def. twist. and bend. aromatic amines, Trp., A, proteins, ring str.,[3-5,10,17,39,51] Amide III, C-N str., N-H bend., skeleton str.,[37] G,[21,33] keratin-8/18 knock-down cells[55] |
| **1340.4** | [9] Amide III, $CH_2$ wag. glycine backbone, Pro. side chain,[43] C-H, $CH_2$ def., C-H bend., $CH_2$ and $CH_3$ twist., wag. and/or bend., C-C str. phenyl and C-O bend., str., in-plane bend., C-N str., C-O-H bend.,Trp. A, G, ring breathing,[1,5,7,10,19,20,22,24,27,28,34,52,67,69,70,89,98] keratin-8/18 knock-down cells[85] |
| **1370.8** | [10] sym. str. $CH_3$, sym. def. $CH_3$, $CH_2$ def., CC def.,[6,52,53], G, Trp.,[4] keratin-8/18 knock-down cells,[55] T, A, G ring breathing modes[1,15] |
| **1439.4** | [11] $CH_3$, $CH_2$, CH def., $CH_2$ sciss., $CH_3$, $CH_2$, C-H bend,[4,6,20,24,28,43,48,52,55,59,61,63,69,71,77,80,93] acyl chains,[27,48] fatty acids,[30,61] |
| **1453.0** | Amide II, C-H, $CH_2$, $CH_3$ asym. bend., $CH_3$, $CH_2$, C-H def., sciss. $CH_2$ str., C-$H_2$ twist., amino acids side chains of the proteins and carbohydrates (vimentin, cytoplasmic region of keratin-8/18 knock-down cells, membrane-cytoplasm-nucleus),[3,6,7,22,26,28,29,34,39,43,52,55,59,64,68,71,80,82,84,91] methyl groups bend., methylene def.,[30] umbrella mode of methoxyl[6,78] |
| **1550.2** | [13] Amide II,[10] Trp., C=C,[4,17,22,41] nucleic acids[24] |
| **1624.8** | Tyr., Phe.,[7] Trp., β-sheet,[3,5,52] C=C olefinic str.,[6,10] C=O str., Amide III[40,74] |
| **1727.4** | C=O str. [74] C=O[6] |
| **2240.6** | [15] -CN,[45] -C≡N,[6] R-C≡C-C≡C-R'[6] |

## REFERENCES


1. Chan, J.W., *et al.* Micro-Raman Spectroscopy Detects Individual Neoplastic and Normal Hematopoietic Cells. *Biophysical Journal* **90**, 648-656 (2006).
2. Peticolas, W.L., Thomas, G.A. & Wang, Y. The Conformation of Duplex of the Dodecamer, d(CGCGAATTCGCG) as a Model for DNA in the liquid state. *Journal of Molecular Liquids* **41**, 367-388 (1989).
3. Salman, A., Shufan, E., Zeiri, L. & Huleihel, M. Detection and identification of cancerous murine fibroblasts, transformed by murine sarcoma virus in culture, using Raman spectroscopy and advanced statistical methods. *Biochimica et Biophysica Acta* **1830**, 2720-2727 (2013).
4. Stone, N., Kendall, C., Smith, J., Crow, P. & Barr, H. Raman spec troscopy for identification of epithelial cancers. *Faraday Discussion.,* **126**, 141– 157 (2004).
5. Cheng, W.-T., Liu, M.-T., Liu, H.-N. & Lin, S.-Y. Micro-Raman spec troscopy used to identify and grade human skin pilomatrixoma. *Microscopy Research and Technique* **68**, 75– 79 (2005).
6. Schulz, H. & Baranka, M. Identification and quantification of valuable plant substances by IR and Ramanspectroscopy. *Vibrational Spectroscopy* **42**, 13-25 (2007).
7. Savoie, R., Jutier, J.-J., Alex, S., Nadeau, P. & Lewis, P.N. Laser Raman Spectra of CALF Thymus Chromatin and its Constituents. *Biophysical Journal* **47**, 451-459 (1985).
8. Peticolas, W.L., Patapoff, T.W., Thomas, G.A., Postlewait, J. & Powell, J.W. Laser Raman Microscopy of Chromosomes in Living Eukaryotic Cells: DNA Polymorphism In Vivo. *Journal of Raman Spectrocopy* **27**, 571-578 (1996).
9. Farguharson, S., Shende, C., Inscore, F.E., Maksymiuk, P. & Gift, A. Analysis of 5-fluorouracil in saliva using surface-enhanced Raman spectroscopy. *Journal of Raman Spectroscopy* **36**, 208– 212 (2005).
10. Keipp, J., Keipp, H., McLaughlin, M., Brown, D. & Keipp, K. *In Vivo* Molecular Probing of Cellular Compartments with Gold Nanoparticles and Nanoaggregates. *Nano Letters* **6**, 2225-2231 (2006).
11. Gniadecka, M., Wulf, H.C., Mortensen, N.N., Nielsen, O.F. & Christensen, D.H. Diagnosis of basal cell carcinoma by Raman spectroscopy. *Journal of Raman Spectroscopy* **28**, 125– 129 (1997).
12. Shetty, G., Kedall, C., Shepherd, N., Stone, N. & Barr, H. Raman spectroscopy: Evaluation of biochemical changes in carcinogenesis of esophagus. *British Journal of Cancer* **94**, 1460– 1464 (2006).
13. Laska, J. & Widlarz, J. Spectroscopic and structural characterization of loe molecular weight fractions of polyaniline. *Polymer* **46**(2005).
14. Thomas, J., G. J. , Prescott, B. & Olins, D.E. Secondary Structure of Histones and DNA in Chromatin. *Science* **197**, 385-387 (1977).
15. Rosenberg-Nicolson, N.L. Probing Nucleosome Core Secondary Structure Before and Afier a-Chymotrypsin Treatment By Raman Spectroscopy and Thermal Denaturation. *Journal of Cellular Biochemistry* **47** 11-17 (1991).
16. Binoy, J., *et al.* NIR-FT Raman and FT-IR spectral studies and ab initio calculations of the anti-cancer drug combretastatin-A4. *Journal of Raman Spectroscopy* **35**, 939– 946 (2004).

17. Stone, N., Kendell, C., Shepherd, N., Crow, P. & Barr, H. Near-infrared Raman spectroscopy for the classification of epithelial pre-cancers and cancers. *Journal of Raman Spectroscopy* **33**, 564– 573 (2002).
18. Movasaghi, Z., Rehman, S. & Rehman, I.U. Raman Spectroscopy of Biological Tissues. *,Applied Spectroscopy Reviews* **42**, 493-541 ( 2007).
19. Notingher, I.*, et al.* Discrimination between ricin and sulphur mustard toxicity in vitro using Raman spectroscopy. *J. R. Soc. Interface* **1**, 79–90 (2004).
20. Wu, H.*, et al.* In vivo lipidomics single-cell Raman spectroscopy *PNAS* **108**, 3809-3814 (2011).
21. Ren, X.*, et al.* Scalable nanolaminated SERS multiwell cell culture assay" Microsystems & Nanoengineering **6**(2020).
22. Hartman, K.A., Clayton, N. & Thomas, J., G. J. . Studies of Virus Structure by Raman Spectroscopy I. R17 Virus and R17 RNA. *Biochemical and Biophysical Research Communications* **50**(1973).
23. Kateinen, E.*, et al.* Quantification of the Amphetamine Content in Seized Street Samples by Raman Spectroscopy. *Journal of Forensic Science* **52**, 88-92 (2007).
24. Knipfer, C.*, et al.* Raman difference spectroscopy: a non-invasive method for identification of oral squamous cell carcinoma. *Biomedical Optics Express* **5**(2014).
25. Kneipp, J., Kneipp, H., Rice, W.L. & Kneipp, K. Optical Probes for Biological Applications Based on Surface-Enhanced Raman Scattering from Indocyanine Green on Gold Nanoparticles. *Anal. Chem.* **77**, 2381-2385 (2005).
26. Tanahashi, K.*, et al.* Assessment of Tumor Cells in a Mouse Model of Diffuse Infiltrative Glioma by Raman Spectroscopy. *BioMed Research International* 1-8 (2014).
27. Shen, N.*, et al.* Steps toward Maturation of Embryonic Stem Cell-Derived Cardiomyocytes by Defined Physical Signals. **9**, 122-135 (2017).
28. Frank, C.J. & McCreary, R.L. Raman Spectroscopy of Normal and Diseased Human Breast Tissues. *Anal. Chem.* **67**, 777-783 (1995).
29. Naumann, D. Infrared and NIR Raman spectroscopy in medical microbiology. in *Proc. SPIE,* , Vol. 3257 245– 257 (1998).
30. Dukor, R.K. Vibrational spectroscopy in the detection of cancer. *Biomedi cal Applications* **5**, 3335– 3359 (2002).
31. Huang, Z., Lui, H., McLean, D.I., Korbelik, M. & Zeng, H. Raman spectroscopy in combination with background near-infrared autofluorescence enhances the *in vivo* assessment of malignant tissues. *Photochemistry and Photobiology* **81**, 1219 –1226 (2005).
32. Lipiec, E.*, et al.* Monitoring UVR induced damage in single cells and isolated nuclei using SR-FTIR microspectroscopy and 3D confocal Raman imaging. *Analyst* **139**, 4200-4209 (2014).
33. Ruiz-Chica, A.J., Medina, M.A., Sanchez-Jimenez, F. & Ramirez, F.J. Characterization by Raman spectroscopy of conformational changes on guanine cytosine and adenine-thymine oligonucleotides induced by aminooxy analogues of spermidine. *Journal of Raman Spectroscopy* **35**, 93 – 100 (2004).
34. Weselucha-Birczynska, A., Kozicki, M., Czepiel, J. & Birczynska, M. Raman micro-spectroscopy tracing human lymphocyte activation. *Analyst* **138**, 7157-7163 (2013).

35. Puppels, G.J., *et al.* Laser Irradiation and RamanSpectroscopy of Single Living Cells and Chromosomes: Sample Degradation Occurs with 514.5 nm but not with 660 nm Laser Light *Experimental Cell Research,* **195**, 361-367 (1991).
36. Sigurdsson, S., *et al.* Detection of skin cancer by classification of Raman spectra. IEEE Transcriptions on Biomedical Engineering. **51**, 10 (2004).
37. Silwal, A.P., Yadav, R., Sprague, J.E. & Lu, H.P. Raman Spectroscopic Signature Markers of Dopamine - Human Dopamine Transporter Interaction in Living Cells. *Neuroscience* (2017).
38. Wood, B.R., Hammer, L., Davis, L. & McNaughton, D. Raman microspectroscopy and imaging provides insights into heme aggregation and denaturation within human erythrocytes. *Journal of Biomedical Optics* **10**(2005).
39. Lau, D.P., *et al.* Raman Spectroscopy for Optical Diagnosis in Normal and Cancerous Tissue of the Nasopharynx—Preliminary Findings. *Lasers in Surgery and Medicine* **32**, 210-214 (2003).
40. Pal, M., *et al.* Iron(III) Complex-Functionalized Gold Nanocomposite as a Strategic Tool for Targeted Photochemotherapy in Red Light. *Inorganic Chemistry* **60**, 6283-6297 (2021).
41. Huang, Z., *et al.* Near-infrared Raman spectroscopy for optical diagnosis of lung cancer. *Inter national Journal of Cancer* **107**, 1047– 1052 (2003).
42. Movasaghi, Z., Rehman, S. & Rehman, I.U. Raman Spectroscopy of Biological Tissues. *Applied Spectroscopy Reviews* **42**, 493-541 (2007).
43. Faolain, E.O., *et al.* A study examining the effects of tissue processing on human tissue sections using vibrational spectroscopy. *Vibrational Spectroscopy* **38**, 121–127 (2005).
44. de Pablo, J.G., *et al.* Detection and time-tracking activation of a photosensitiser on live single colorectal cancer cells using Raman spectroscopy. *The Royal Society of Chemistry, Analyst* **145**, 5878–5888 (2020).
45. Wang, J., Liang, D., Feng, J. & Tang, X. Multicolor Cocktail for Breast Cancer Multiplex Phenotype Targeting and Diagnosis Using Bioorthogonal Surface-Enhanced Raman Scattering Nanoprobes. *Anal. Chem.* **91**, 11045-11054 (2019).
46. Zhang, L. & Min, W. Bioorthogonal chemical imaging of metabolic changes during epithelial–mesenchymal transition of cancer cells by stimulated Raman scattering microscopy. *J Biomed Opt* **22**, e106010 106011- 106017 (2017).
47. Yu, Y., Ramachandran, P.V. & Wang, M.C. Shedding new light on lipid functions with CARS and SRS microscopy. *Biochimica et Biophysica Acta* **1841** 1120-1129 (2014).
48. Krafft, C., Neudert, L., Simat, T. & Salzer, R. Near infrared Raman spectra of human brain lipids. *Spectrochimica Acta, Part A,* **61**, 1529– 1535 (2005).
49. Mordechai, S., Sahu, R.K. & Hammody, Z. Possible common bio markers from FTIR microspectroscopy of cervical cancer and melanoma. *Journal of Microscopy* **215** 86 – 91 (2004).
50. Andrus, P.G.L. & Strickland, R.D. Cancer grading by Fourier transform infrared spectroscopy. *Biospectroscopy* **4**, 37 – 46 (1998).
51. Marro, M., Nieva, C., Sanz-Pamplona, R. & Sierra, A. Molecular monitoring of epithelial-to-mesenchymal transition in breast cancer cells by means of Raman spectroscopy. *Biochimica Biophysica Acta* **1843**, 1785 – 1795 (2014).
52. Lakshmi, R.J., *et al.* Tissue Raman Spectroscopy for the Study of Radiation Damage: Brain Irradiation of Mice. *Radiation Research* **157**, 175-182 (2002).

53. Depciuch, J.*, et al.* Correlation between human colon cancer specific antigens and Raman spectra. Attempting to use Raman spectroscopy in the determination of tumor markers for colon cancer. *Nanomedicine: Nanotechnology, Biology, and Medicine* **48**, 102657-102656 (2023).
54. Rix, J.*, et al.* Correlation of biomechanics and cancer cell phenotype by combined Brillouin and Raman spectroscopy of U87-MG glioblastoma cells. *J. R. Soc. Interface* **19**, 0209 pp.0201-0211 (2022).
55. Singh, S.P.*, et al.* Identification of morphological and biochemical changes in keratin-8/18 knock-down cells using Raman spectroscopy. *J. Biophotonics* 1-8 (2017).
56. Min, Y.-K., Yamamoto, T., Kohoda, E., Ito, T. & Hamaguchi, H. 1064 nm near-infrared multichannel Raman spectroscopy of fresh human lung tissues. *Journal of Raman Spectroscopy* **36**, 73 – 76 (2005).
57. Huang, P.-J.*, et al.* Surface-enhanced Raman scattering (SERS) by gold nanoparticle characterizes dermal thickening by collagen in bleomycin-treated skin *ex vivo*. *Skin Res Technology* e13334(pp. 13331-13338) (2023).
58. Ahmed, S., Baijal, G., Somashekar, R., Iyer, S. & Nayak, V. One Pot Synthesis of PEGylated Bimetallic Gold–Silver Nanoparticles for Imaging and Radiosensitization of Oral Cancers. *International Journal of Nanomedicine* **16**, 7103-7121 (2021).
59. Malini, R., Venkatakrishma, K. & Kurien, J. Discrimination of normal, inflammatory, premalignant, and malignant oral tissue: A Raman spectroscopy study. *Biopolymers* **81** 179–193 (2006).
60. Shapiro, A., Gofrit, O.N., Pizov, G., Cohen, J.K. & Maier, J. Raman Molecular Imaging: A Novel Spectroscopic Technique for Diagnosis of Bladder Cancer in Urine Specimens. *European Urology* **59**, 162-112 (2011).
61. Hanlon, E.B.*, et al.* Prospects for in vivo Raman spectroscopy. *Physics in Medicine and Biology* **45**, 1– 59 (2000).
62. Utzinger, U.R.S., Heintzelman, D.L. & Mahadevan-Jansen, A. Near infrared Raman spectroscopy for in vivo detection of cervical precancers. *Applied Spectroscopy* **55** 955–959 (2001).
63. Kolijenovic, S., Scut, T.B., Vincent, A., Kros, J.M. & Puppels, G.J. Detection of meningioma in dura mater by Raman spectroscopy. *Analytical Chemistry* **77** 7958– 7965 (2005).
64. Som, D.*, et al.* A grid matrix-based Raman spectroscopic method to characterize different cell milieu in biopsied axillary sentinel lymph nodes of breast cancer patients. *Lasers Med. Sci.* (2015).
65. Yeu, T.H.*, et al.* Distinct Lipid Phenotype of Cancer-Associated Fibroblasts (CAFs) Isolated From Overweight/Obese Endometrial Cancer Patients as Assessed Using Raman Spectroscopy. *Applied Spectroscopy* **77**, 723-733 (2023).
66. Katainen, E.*, et al.* Quantification of the Amphetamine Content in Seized Street Samples by Raman Spectroscopy. *Journal of Forensic Science* **52**, 88-92 (2007).
67. Manciu, F.S.*, et al.* Assessing Nordihydroguaiaretic Acid Therapeutic Effect for Glioblastoma Multiforme. *Sensors* **22**(2022).
68. De Luca, A.C., Mazilu, M., Riches, A., Herrington, C.S. & Dholakia, K. Online Fluorescence Suppression in Modulated Raman Spectroscopy. *Anal. Chem.* **82**, 738–745 (2010).

69. Mahadevan-Jansen, A. & Richards-Kortum, R. Raman spectroscopy for cancer detection. in *19th Int. Conf. IEEE EMBS* (Chicago, 1997).
70. Fung, M.F.K., Senterman, M.K., Mikhael, N.Z., Lacelle, S. & Wong, P.T.T. Pressure-tuning Fourier transform infrared spectroscopic study of carcinogenesis in human endometrium. Biospectroscopy. **2**, 155– 165 (1996).
71. Silveira, L., Sathaiah, S. & Zangaro, R.A. Correlation between near infrared Raman spectroscopy and the histopathological analysis of atherosclerosis in human coronary arteries. *Lasers in Surgery and Medicine* **30**, 290– 297 (2002).
72. Wood, B.R*., et al.* FTIR microspectroscopic study of cell types and potential confounding variables in screening for cervical malignancies. *Biospec troscopy*, 75 – 91 (1998).
73. Scarpitti, B.T., Morrison, A.M., Buyanova, M. & Schultz, Z.D. Comparison of 4-Mercaptobenzoic Acid SERS Based Methods for pH Determination In Cells. *Appl Spectrosc.* **74**, 1423-1432 (2020).
74. Shaw, R.A. & Mantsch, H.H. Vibrational biospectroscopy: From plants to animals to humans. A historical perspective. *Journal of Molecular Structure,* **480– 481**, 1– 13 (1999).
75. Deng, J.L., Wei, Q., Zhang, M.H., Wang, Y.Z. & Li, Y.Q. Study of the effect of alcohol on single human red blood cells using near-infrared laser tweezers Raman spectroscopy. *Journal of Raman Spectroscopy* **36**, 257– 261 (2005).
76. Huang, Z*., et al.* Effect of formalin fixation on the near-infrared Raman spec troscopy of normal and cancerous human bronchial tissues. . *International Journal of Oncology* **23**, 649– 655 (2003).
77. Lau, D.P*., et al.* Raman spectroscopy for optical diagnosis in the larynx: Preliminary findings. *Lasers in Surgery and Medicine* **37**, 192– 200 (2005).
78. Choo-Smith, L.-P*., et al.* Medical applications of Raman spectroscopy: From proof of principle to clinical implementation. *Biopolymers (Biospectroscopy)* **67**, 1 – 9 (2002).
79. Kaminaka, S., Yamazaki, H., Ito, T., Kohoda, E. & Hamaguchi, H. Near infrared Raman spectroscopy of human lung tissues: Possibility of molecular-level cancer diagnosis. *Journal of Raman Spectroscopy* **32**, 139– 141 (2001).
80. Shafer-Peltier, K.E*., et al.* Raman microspectroscopic model of human breast tissue: Impli cations for breast cancer diagnosis in vivo. *Journal of Raman Spectroscopy* **33**, 552– 563 (2002).
81. Short, M.A*., et al.* Changes in nuclei and peritumoral collagen within nodular basal cell carcinomas via confocal micro-Raman spectroscopy. *Journal of Biomedical Optics* **11** 34004 –34013 (2006).
82. Tan, Y.-Y*., et al.* Design of auto-classifying system and its application in Raman spectroscopy diagnosis of gastric carcinoma. in *2nd Inter. Conf. Machine Learning & Cybernetics* (2003).
83. Viehoever, A.R., Anderson, D., Jansen, D. & Mahadevan-Jansen, A. Organotypic raft cultures as an effective in vitro tool for understanding Raman spectral analysis of tissues. *Photochemistry and Photobiology* **78** 517– 524 (2003).
84. Fleissner, F*., et al.* Tension Causes Unfolding of Intracellular Vimentin Intermediate Filaments. *Advanced Biosystems* **4** 2000111 -20001112 (2020).
85. Singh, C*., et al.* Entropy-Mediated Patterning of Surfactant-Coated Nanoparticles and Surfaces. *Physical Review Letters* **99**, 1-3 (2007).

86. Farahavar, G.*, et al.* Single-chain antibody-decorated Au nanocages@liposomal layer nanoprobes for targeted SERS imaging and remote-controlled photothermal therapy of melanoma cancer cells. *Materials Science & Engineering C* **124**, 112086 pp. 112081-112011 (2021).
87. Seballos, L., Zhang, J.Z. & Sutphen, R. Surface-enhanced Raman scattering detection of lysophosphatidic acid. *Analytical and Bioanalytical Chemistry* **383**, 763–767 (2005).
88. Sebag, J., Nie, S., Reiser, K. & Yu, N.-T. Raman spectroscopy character ization of diabetes effects on human vitreous in diabetic retinopathy in *Proceedings of the SPIE*, Vol. 1877 284–288 (1993).
89. Puppeles, G.J., Garritsen, H.S.P., Kummer, J.A. & Greve, J. Carotenoids located in human lymphocyte subpopulations and natural killer cells by Raman microspectroscopy. *Cytometry* **14**, 251– 256 (1993).
90. Strobbia, P.*, et al.* Accurate in vivo tumor detection using plasmonicenhanced shifted-excitation Raman difference spectroscopy (SERDS). *Theranostics* **11**, 4090-4102 (2021).
91. Kaminaka, S., Ito, T., Yamazaki, H., Kohoda, E. & Hamaguchi, H. Near infrared multichannel Raman spectroscopy toward real-time in vivo cancer diagnosis. *Journal of Raman Spectroscopy* **33**, 498– 502 (2002).
92. Christiofori, G. & Semb, H. The role of cell-adhesion molecule E-cadherin as a tumor suppressor gene. *Trends Biochem. Sci.* **24**, 73-76 (1999).
93. Lipiec, E.*, et al.* Monitoring UVR induced damage in single cells and isolated nuclei using SR-FTIR microspectroscopy and 3D confocal Raman imaging. *Analyst* **139** 4200-4209 (2014).
94. Kachrimanis, K., Braun, D.B. & Griesser, U.J. Quantitative analysis of paracetamol polymorphs in powder mixtures by FT-Raman spectroscopy and PLS regression. *Journal of Pharmaceutical and Biomedical Analysis* **43**, 407– 412 (2007).
95. Lucassen, G.W., Van Veen, G.N. & Jansen, J.A. Band analysis of hydrated human skin stratum corneum attenuated total reflectance Fourier transform infrared spectra in vivo. *Journal of Biomedical Optics* **3**, 267– 280 (1998).
96. Zimmer, G., Günther, H.O. & Schmidt, H. "Interaction of Phloretin with the Human Red Cell Membrane and Membrane Lipids: Evidence from Infrared, Raman and ESR Spectroscopy. *Z. Naturforsch.* **36 c**, 586-592 (1981).
97. Dovbeshko, G.I., Gridina, N.Y., Kruglova, E.B. & Pashchuk, O.P. FTIR spectroscopy studies of nucleic acid damage. *Talanta* **53**, 233–246 (2000).
98. Singha, A., Ghosh, A., Roy, A. & Ray, N.R. Quantitative avalysis of hydrogenated diamondlike carbon films by visible Raman spectroscopy. *Journal of Applied Physics* **100** 1 – 8 (2006).
